# Serotonin drives the acquisition of a profibrotic and anti-inflammatory gene profile through the 5-HT7R-PKA signaling axis

**DOI:** 10.1038/s41598-017-15348-y

**Published:** 2017-11-07

**Authors:** Ángeles Domínguez-Soto, Alicia Usategui, Mateo de las Casas-Engel, Miriam Simón-Fuentes, Concha Nieto, Víctor D. Cuevas, Miguel A. Vega, José Luis Pablos, Ángel L. Corbí

**Affiliations:** 1 Myeloid Cell Laboratory, Centro de Investigaciones Biológicas, CSIC, Madrid, Spain; 2 Servicio de Reumatología, Instituto de Investigación Hospital 12 de octubre, Universidad Complutense de Madrid, Madrid, Spain

## Abstract

Peripheral serotonin (5-hydroxytryptamine, 5-HT) regulates cell growth and differentiation in numerous cell types through engagement of seven types of cell surface receptors (HTR1–7). Deregulated 5-HT/HTR levels contribute to pathology in chronic inflammatory diseases, with macrophages being relevant targets for the physio-pathological effects of 5-HT. In fact, 5-HT skews human macrophage polarization through engagement of 5-HT2BR and 5-HT7R receptors. We now report that 5-HT primes macrophages for reduced pro-inflammatory cytokine production and IFN type I-mediated signaling, and promotes an anti-inflammatory and pro-fibrotic gene signature in human macrophages. The acquisition of the 5-HT-dependent gene profile primarily depends on the 5-HT7R receptor and 5-HT7R-initiated PKA-dependent signaling. In line with the transcriptional results, 5-HT upregulates TGFβ1 production by human macrophages in an HTR7- and PKA-dependent manner, whereas the absence of *Htr7 in vivo* results in diminished macrophage infiltration and collagen deposition in a mouse model of skin fibrosis. Our results indicate that the anti-inflammatory and pro-fibrotic activity of 5-HT is primarily mediated through the 5-HT7R-PKA axis, and that 5-HT7R contributes to pathology in fibrotic diseases.

## Introduction

Serotonin (5-hydroxytryptamine, 5-HT) is a monoamine neurotransmitter derived from L-tryptophan via a rate-limiting reaction catalyzed by tryptophan hydroxylases (TPH1 in periphery, TPH2 in brain)^1,2^. Brain-derived 5-HT controls mood, behavior, sleep, blood pressure and thermoregulation^3^ whereas peripheral 5-HT regulates vascular and heart functions^4^ and gastrointestinal mobility^5^. Enterochromaffin cells in the gastrointestinal tract produce 90% of the human body’s 5-HT^6^, which is actively taken up by blood platelets and stored in dense granules. Upon platelet activation, released 5-HT modifies vascular smooth muscle tone, promotes proliferation of smooth muscle cells^7^, hepatocytes^8^ and endothelial cells^9^, and critically contributes to wound healing. All 5-HT actions are exerted through engagement of seven types of receptors (5-HT1-7R) which, except for 5-HT3R, belong to the G protein-coupled superfamily of receptors^10^. 5-HT also functions as a regulator of immune and inflammatory responses^11^. 5-HT modulates T-cell activation, proliferation and differentiation^12^ and modifies cytokine production in a cell type-dependent manner^13–16^. The regulatory role of 5-HT in inflammation is illustrated by the pathological consequences of its altered production or absence in chronic inflammatory diseases. 5-HT contributes to Pulmonary Arterial Hypertension (PAH)^17^, atopic dermatitis^18^ and systemic sclerosis^19^, and modifies the outcome of inflammatory gut disorders^20–25^. 5-HT also favors colon cancer angiogenesis^26^ and neuroendocrine neoplasms proliferation^27^, and its absence increases pathologic scores in collagen-induced arthritis^28^. The close link between 5-HT and chronic inflammatory pathologies^29^ is in line with the anti-inflammatory actions of selective 5-HT reuptake inhibitors (SSRI) like fluoxetine^30,31^. Further supporting the 5-HT/inflammation link, 5-HT2BR has been shown to mediate the effects of 5-HT on tissue fibrosis^19^ and PAH^17^, 5-HT7R mediates the 5-HT contribution to gut inflammation in IBD models^20,22,23^, and 5-HT3R or 5-HT4R ligands reduce inflammatory reactions during postoperative ileus^32,33^.

Macrophages are critical for maintaining tissue homeostasis and promoting the initiation and resolution of inflammatory processes. The balance between pro- and anti-inflammatory (resolving) macrophages is required for restoring tissue homeostasis^34–36^, and its deregulation leads to chronic inflammatory diseases^37,38^. Since macrophages rapidly adapt their functions to micro-environmental stimuli (e.g., cytokines, growth factors, pathogen- and damage-associated molecular patterns)^34–38^, targeting macrophages is currently proposed as a therapeutic approach for chronic inflammatory diseases^37^. Not surprisingly, some of the effects of 5-HT on inflammation are mediated through direct and indirect actions on myeloid cells^17,26,32,33^. Further supporting this relationship, bone marrow-derived cells are responsible for the contribution of 5-HT and 5-HT7R to intestinal inflammation^22^, and macrophages mediate the anti-inflammatory action of SSRI^30,39^.

We have previously demonstrated that human pro-inflammatory and anti-inflammatory macrophages^40–43^ exhibit a distinct profile of 5-HT receptors, and that 5-HT2BR and 5-HT7R shape macrophage effector functions towards the anti-inflammatory side^44^. To dissect the molecular mechanisms underlying the inflammation-modulating action of 5-HT, we undertook the determination of the 5-HT-dependent transcriptome of human macrophages. 5-HT rapidly altered the human macrophage transcriptome towards a growth-promoting, anti-inflammatory and pro-fibrotic gene profile, whose acquisition was dependent on the 5-HT7R -PKA signaling axis. Moreover, and in line with these findings, *Htr7* ^−/−^ mice exhibited significantly reduced macrophage accumulation and collagen deposition in a bleomycin-induced model of skin fibrosis.

## Results

### 5-HT promotes the expression of an anti-inflammatory gene profile and inhibits pro-inflammatory cytokine production

**Figure 1:**
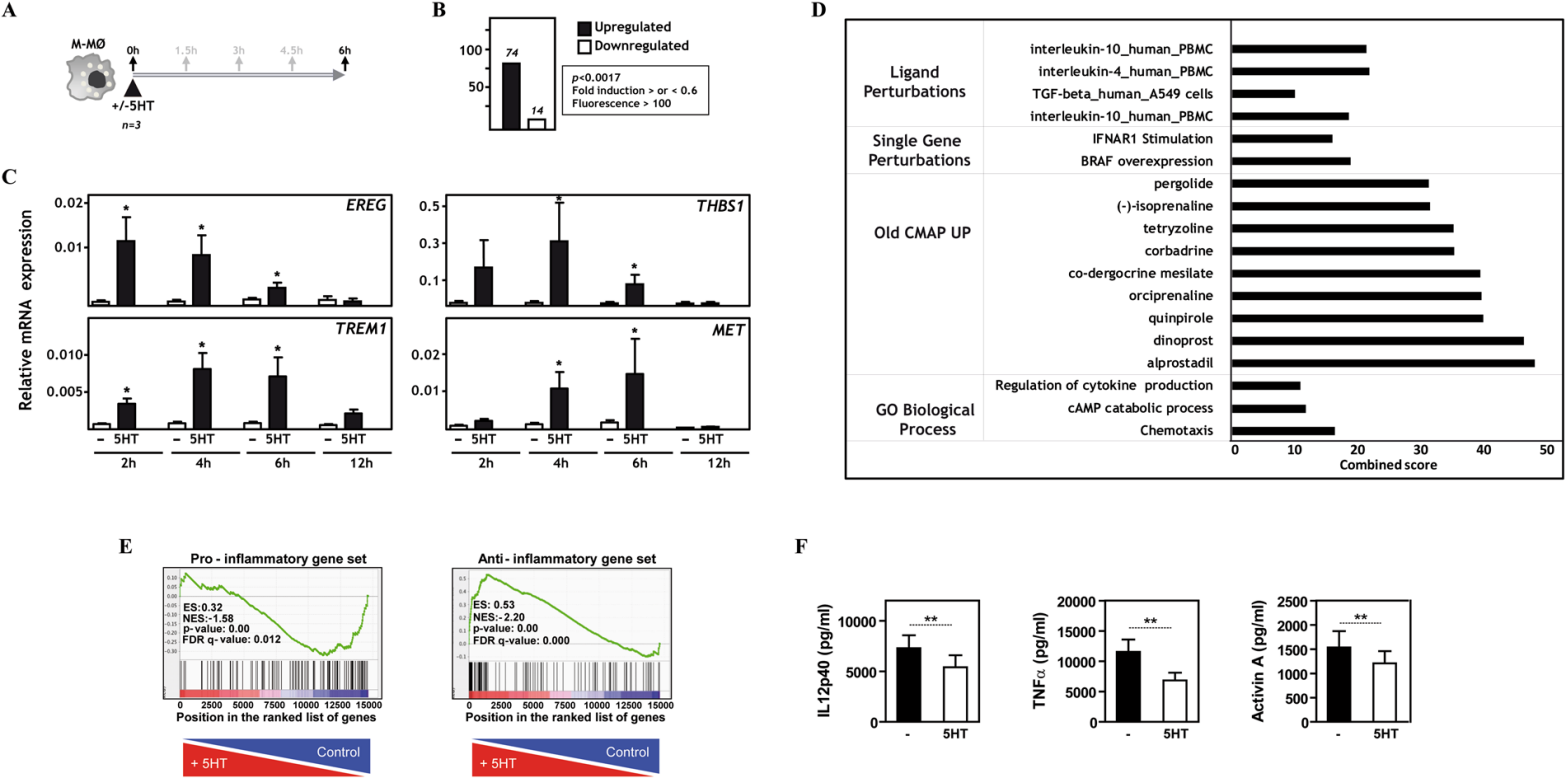
Serotonin promotes the acquisition of an anti-inflammatory gene profile and conditions human macrophages for diminished LPS-stimulated proinflammatory cytokine production. (**A**) Experimental design of the gene profiling experiment. (**B**) Number of annotated genes whose expression is upregulated or downregulated in M-MØ exposed to 5-HT for 6 hours (p < 0.0017; Upregulated, log2 M-MØ + 5-HT/M-MØ > 0.6; Downregulated, log2 M-MØ + 5-HT/M-MØ < −0.6). (**C**) Relative expression of the indicated genes in non-treated M-MØ (-) or M-MØ treated with 5-HT (5HT) for 2, 4, 6 and 12 hours. Results are expressed as the mRNA level of each gene relative to the *GAPDH* mRNA level in the same sample (n = 3; *p < 0.05). (**D**) Gene ontology analysis of the set of genes upregulated by 5-HT in M-MØ, as determined using the ENRICHR tool and the indicated databases [combined score = log(p-value) x z-score]. (**E**) GSEA on the “t statistic-ranked” list of genes obtained from the 5-HT-treated M-MØ versus M-MØ limma analysis, using the proinflammatory (left panel) and anti-inflammatory (right panel) gene sets previously defined^48^. Vertical black lines indicate the position of each of the genes comprising the “Pro-inflammatory” and “Anti-inflammatory” gene sets. (**F**) Production of IL-12p40, TNFα and Activin A by non-treated (-) or 5-HT-pretreated (6 h) LPS-stimulated (18 h) M-MØ, as determined by ELISA (n = 12; *p < 0.05; **p < 0.01).

To determine the 5HT-dependent transcriptome of human macrophages, a global gene expression analysis was performed on human monocyte-derived macrophages (M-MØ) exposed to 5-HT for 6 hours (Fig. 1A), a time at which exposure to 5-HT modifies the LPS-induced production of inflammatory cytokines (*see below*). Transcriptional profiling revealed that 5-HT increases the expression of 170 annotated genes (p < 0.01; log_2_ ratio 5-HT/untreated ≥0.6) and downregulated the expression of 41 genes (p < 0.01; log_2_ ratio 5-HT/untreated ≤ −0.6) (Supplementary Table I). Further filtering (normalized expression levels higher than 100 in untreated or 5-HT-treated M-MØ, and p < 0.0017 for the M-MØ vs. M-MØ + 5-HT comparison) identified 74 genes upregulated and 14 genes downregulated upon 5-HT exposure (Table 1) (Fig. 1B). Analysis of independent 5-HT-treated M-MØ samples confirmed the microarray data and revealed distinct kinetics for the 5-HT-upregulated genes. As shown in Fig. 1C, *EREG* expression was maximally upregulated only two hours after exposure to 5-HT, while other genes (*TREM1, MET*, *THBS1*) exhibited maximal level of up-regulation 4–6 hours after 5-HT treatment. Therefore, 5-HT modifies the gene signature of human macrophages and its effects can be detected as early as 2 hours after exposure to the neurotransmitter.

**Table 1:** Gene expression analysis on untreated (M-MØ) and 5-HT-treated (10 μM, 6h) M-MØ (M-MØ+5HT), where the normalized fluorescence of each probe (*probeid*) and the corresponding gene symbol (*genesymbol*) is indicated. Probes are ordered according to the t-value (*t*) of the M-MØ+5HT/ M-MØ ratio, and the p values obtained after adjusting for multiple hypotheses testing (*adj.pval*) are shown.

probeid	M-MØ	M-MØ + 5HT	log2.fold M-MØ + 5HT/M-MØ	t	pval	adj.pval	genesymbol
A_23_P19333	80.05	344.74	2.0767	16.5371	0	0.192	TREM1
A_23_P401106	154.79	2069.16	3.6833	14.6249	0	0.192	PDE2A
A_23_P416711	51.15	137.60	1.4367	14.5716	0	0.192	ST6GALNAC3
A_23_P208900	275.45	686.62	1.3333	14.3717	0	0.192	SEMA6B
A_23_P32404	410.56	963.29	1.2367	13.1219	1.0000E-04	0.2092	ISG20
A_23_P132515	102.95	300.13	1.55	12.9583	1.0000E-04	0.2092	SIDT1
A_23_P257649	331.87	745.56	1.1767	12.2964	1.0000E-04	0.2138	RBP1
A_23_P373724	55.60	115.94	1.0967	11.7738	1.0000E-04	0.2138	PPFIBP1
A_24_P49260	120.84	254.30	1.14	11.5085	1.0000E-04	0.2138	SPTLC3
A_23_P154605	3414.30	7447.51	1.1567	10.6682	2.0000E-04	0.2138	SULF2
A_23_P85453	45.04	102.33	1.2633	10.2964	2.0000E-04	0.2138	CD244
A_24_P32085	269.48	651.45	1.2633	10.2497	2.0000E-04	0.2138	MOB3B
A_33_P3352578	74.65	140.30	0.93	10.043	2.0000E-04	0.2138	CLEC4D
A_23_P321354	389.53	744.31	0.9467	10.037	2.0000E-04	0.2138	TMEM71
A_33_P3333436	78.19	169.81	1.1033	9.9911	2.0000E-04	0.2138	SGSM3
A_23_P69109	1470.47	2684.51	0.8667	9.957	2.0000E-04	0.2138	PLSCR1
A_24_P349547	218.04	750.66	1.9733	9.8847	2.0000E-04	0.2138	
A_33_P3322288	101.13	372.80	1.95	9.6901	2.0000E-04	0.2138	AZI2
A_24_P132383	491.79	927.06	0.94	9.3149	3.0000E-04	0.2138	GIMAP8
A_24_P93703	371.19	656.60	0.8167	8.9764	3.0000E-04	0.2138	TMEM198B
A_24_P324674	492.95	846.92	0.7767	8.9556	3.0000E-04	0.2138	LY9
A_23_P414654	56.39	113.19	0.9633	8.9112	3.0000E-04	0.2138	RAB37
A_24_P245379	51.84	148.73	1.4133	8.8935	3.0000E-04	0.2138	SERPINB2
A_23_P72117	446.16	1126.78	1.2967	8.8485	4.0000E-04	0.2138	SMPDL3A
A_24_P296508	702.06	1395.79	0.9533	8.7928	4.0000E-04	0.2138	SLC43A2
A_32_P161762	69.56	124.60	0.8667	8.7471	4.0000E-04	0.2138	RUNX2
A_23_P166297	227.26	450.70	0.9833	8.6544	4.0000E-04	0.2138	ABCG1
A_23_P428129	407.37	1022.43	1.32	8.6251	4.0000E-04	0.2138	CDKN1C
A_23_P102731	127.71	316.19	1.2833	8.6233	4.0000E-04	0.2138	SMOX
A_23_P404481	116.49	243.45	1.1133	8.5894	4.0000E-04	0.2138	S1PR1
A_33_P3226050	548.01	953.06	0.8	8.4776	4.0000E-04	0.2138	GATSL3
A_23_P10559	264.88	447.64	0.75	8.4521	4.0000E-04	0.2138	AATK
A_33_P3240843	170.64	314.95	0.8833	8.4384	4.0000E-04	0.2138	TMEM71
A_33_P3382560	7622.06	15383.62	1.06	8.424	4.0000E-04	0.2138	RPL23A
A_23_P25503	2952.57	6154.31	1.0633	8.4164	5.0000E-04	0.2138	FNDC3A
A_23_P86653	11484.56	19458.03	0.77	8.3019	5.0000E-04	0.2193	SRGN
A_23_P15108	532.44	913.10	0.77	8.1222	5.0000E-04	0.2193	YPEL3
A_23_P37375	217.10	359.90	0.7433	8.0574	5.0000E-04	0.2193	RPS6KA5
A_23_P89570	551.51	948.42	0.7633	8.0314	6.0000E-04	0.2193	ZMYND15
A_33_P3236177	703.17	1139.93	0.6967	7.9894	6.0000E-04	0.2193	ANG
A_23_P170453	4108.14	10205.73	1.4933	7.968	6.0000E-04	0.2193	CST5
A_33_P3273885	409.11	870.16	1.0067	7.9645	6.0000E-04	0.2193	
A_33_P3323722	2078.85	4415.23	1.0967	7.9589	6.0000E-04	0.2193	ARL4C
A_23_P59637	957.90	1874.73	0.96	7.9163	6.0000E-04	0.2193	DOCK4
A_23_P154849	87.31	145.08	0.7267	7.9088	6.0000E-04	0.2193	OLIG1
A_23_P167920	117.43	211.91	0.8467	7.7999	6.0000E-04	0.2296	DLL1
A_24_P315184	9659.77	16932.61	0.8333	7.7041	7.0000E-04	0.2387	NBEAL1
A_32_P117464	190.00	365.73	0.97	7.6492	7.0000E-04	0.2392	MB21D2
A_33_P3422294	101.01	288.34	1.4233	7.6364	7.0000E-04	0.2392	
A_33_P3317431	1153.70	2714.24	1.3067	7.5237	8.0000E-04	0.2392	
A_23_P110445	129.59	225.31	0.8067	7.5107	8.0000E-04	0.2392	APBB3
A_23_P23438	741.59	1400.56	0.9633	7.4906	8.0000E-04	0.2392	SEMA4A
A_23_P57760	101.41	159.07	0.6567	7.4768	8.0000E-04	0.2392	ACPL2
A_33_P3351775	79.06	130.10	0.6867	7.4673	8.0000E-04	0.2392	
A_33_P3227716	820.46	1369.66	0.7333	7.4591	8.0000E-04	0.2392	GATSL3
A_23_P76969	785.27	2508.70	1.6	7.4097	8.0000E-04	0.2392	SIPA1L1
A_23_P115011	59.66	102.35	0.8333	7.3499	8.0000E-04	0.2392	ADAMTSL4
A_32_P87697	29475.87	46802.86	0.6833	7.3421	8.0000E-04	0.2392	HLA-DRA
A_23_P55356	279.95	489.77	0.82	7.2414	9.0000E-04	0.2392	VMO1
A_23_P214139	349.66	647.84	0.8833	7.2258	9.0000E-04	0.2392	REV3L
A_33_P3259557	296.93	544.75	0.8333	7.1249	0.001	0.2435	TMEM198B
A_23_P79518	166.63	375.23	1.0967	7.111	0.001	0.2435	IL1B
A_24_P370172	236.53	391.70	0.72	7.0777	0.001	0.2457	LILRA5
A_23_P152791	163.03	270.51	0.75	6.851	0.0011	0.2668	SLC16A6
A_23_P400378	512.19	844.84	0.8067	6.8421	0.0011	0.2668	GPBAR1
A_23_P85716	2690.03	4201.95	0.6933	6.8414	0.0011	0.2668	FCGR2A
A_33_P3413840	121.69	228.70	0.9067	6.8214	0.0012	0.2668	GK
A_33_P3372727	54.20	141.80	1.44	6.8087	0.0012	0.2668	SEMA5A
A_23_P38795	1232.70	2077.44	0.8033	6.7376	0.0012	0.2692	FPR1
A_33_P3352098	11435.65	17831.64	0.6367	6.7192	0.0012	0.2692	MS4A7
A_33_P3315320	70.38	106.96	0.6	6.7149	0.0012	0.2692	CNTD1
A_23_P55020	656.97	1013.28	0.6133	6.659	0.0013	0.2725	CD300LF
A_33_P3415191	182.14	313.76	0.78	6.6105	0.0013	0.2733	ATP8B1
A_33_P3232557	119.84	206.66	0.7833	6.5667	0.0014	0.2733	DLGAP3
A_23_P41114	878.89	1323.40	0.6167	6.4491	0.0015	0.2733	CSTA
A_23_P201747	114.55	205.24	0.7933	6.4307	0.0015	0.2733	PADI2
A_33_P3236267	228.15	690.87	1.7367	6.4271	0.0015	0.2733	KCNQ1OT1
A_23_P380998	165.10	269.18	0.74	6.4024	0.0015	0.2733	R3HDM1
A_23_P60166	395.34	627.24	0.6367	6.3945	0.0016	0.2733	DEPTOR
A_23_P215744	91.56	144.62	0.7067	6.3883	0.0016	0.2733	CTTNBP2
A_23_P258108	11895.64	19363.83	0.7267	6.3718	0.0016	0.2733	
A_33_P3386132	397.26	623.42	0.66	6.3502	0.0016	0.2733	C2orf49
A_24_P110273	676.36	1044.92	0.6333	6.3217	0.0016	0.2733	
A_23_P25566	4726.99	7713.17	0.7067	6.3212	0.0016	0.2733	GPR183
A_23_P67896	269.49	137.51	−0.9233	−6.3894	0.0016	0.2733	SCN3A
A_23_P125204	492.06	317.00	−0.6133	−6.4729	0.0015	0.2733	OR10G8
A_33_P3232955	920.03	555.00	−0.77	−6.662	0.0013	0.2725	F2RL3
A_32_P134290	1591.48	1022.82	−0.65	−6.7639	0.0012	0.2689	ZCCHC2
A_23_P62967	1743.65	1144.21	−0.6033	−6.7737	0.0012	0.2689	DISC1
A_33_P3512350	675.48	399.65	−0.7567	−6.9789	0.0011	0.2531	LOC339807
A_23_P340848	1536.35	979.80	−0.6667	−7.0574	0.001	0.246	PTGIR
A_23_P103034	214.84	114.56	−0.9067	−7.2742	9.0000E-04	0.2392	CRYBA4
A_23_P328545	440.40	259.98	−0.7633	−8.0807	5.0000E-04	0.2193	GABRP
A_23_P404162	100.99	46.26	−1.0767	−8.2829	5.0000E-04	0.2193	HDAC9
A_23_P81441	1493.93	874.24	−0.76	−8.7105	4.0000E-04	0.2138	C5orf20
A_33_P3380383	4374.93	2579.19	−0.8067	−8.8739	4.0000E-04	0.2138	TIFAB
A_23_P91095	523.44	291.00	−0.8367	−9.6298	2.0000E-04	0.2138	CD28
A_32_P155666	716.76	350.13	−1.0733	−9.703	2.0000E-04	0.2138	ECEL1

Gene ontology analysis supported the relevance of the transcriptomic data because the 5-HT-upregulated gene set included a significant percentage of genes whose expression is increased by serotonin receptors agonists like co-dergocrine mesilate (adj *p* = 2.9 × 10^−10^) and pergolide (adj *p* = 3.03 × 10^−8^) (Fig. 1D). In fact, pergolide is an agonist for 5-HT2BR that causes valvular heart disease^45,46^ and M-MØ express functional 5-HT2BR receptors^44^. Besides, 5-HT enhanced the expression of genes positively regulated by prostaglandins (Alprostidil, Dinoprost), dopamine (Quinpirole, co-dergocrine mesilate), and adrenergic (Orciprenaline, Tetryzoline, (-)-isoprenaline) receptor ligands (Fig. 1D). Regarding biological processes, gene ontology analysis revealed that 5-HT-upregulated genes are significantly enriched in genes involved in chemotaxis (adj *p* = 1.8 × 10^−3^), cAMP catabolic process (adj *p* = 1.3 × 10^−2^) and regulation of cytokine production (adj *p* = 1.7 × 10^−2^), as well as in genes regulated by IL-10 (adj *p* = 8.3 × 10^−6^ and adj *p* = 4.2 × 10^−5^) and TGFβ (adj *p* = 2.7 × 10^−3^), and negatively regulated upon IFNAR1 stimulation (adj *p* = 7.0 × 10^−5^) (Fig. 1D).

The availability of the 5-HT-dependent transcriptome in M-MØ allowed us to address the global effect of 5-HT on the macrophage transcriptome. We have previously determined the gene expression profiles of IL-10-producing anti-inflammatory (M-MØ) and TNF-α-producing pro-inflammatory (GM-MØ) human macrophages^47–49^, and identified two sets of 150 genes that best define their corresponding transcriptomes (“Anti-inflammatory gene set” for M-MØ, and “Pro-inflammatory gene set” for GM-MØ)^47,48^. Analysis of the expression of both gene sets in 5-HT-treated macrophages using Gene Set Enrichment Analysis (GSEA) revealed that, compared to untreated cells, the transcriptome of 5-HT-treated macrophages shows a significantly higher expression of the “Anti-inflammatory gene set” (FDR q value = 0.000) together with a significantly lower expression of the “Pro-inflammatory gene set” (FDR q value = 0.012) (Fig. 1E). In fact, genes within the leading edge of the “anti-inflammatory gene set” included *CD163L1*, *HTR2B*, and *IL10*, whose expression is closely linked to M-CSF-driven anti-inflammatory polarization^44,48,49^ (Fig. 1E, right panel and data not shown). At the functional level, and in line with GSEA results, 5-HT pretreatment (6 hours) led to a significant reduction in the LPS-induced production of IL-12p40, TNFα and Activin A of M-MØ (Fig. 1F). Therefore, 5-HT promotes the acquisition of an anti-inflammatory gene profile and conditions macrophages for a diminished pro-inflammatory response towards pathogenic stimuli.

### 5-HT modulates macrophage transcriptome and function primarily via the 5-HT7R-PKA signaling axis

**Figure 2:**
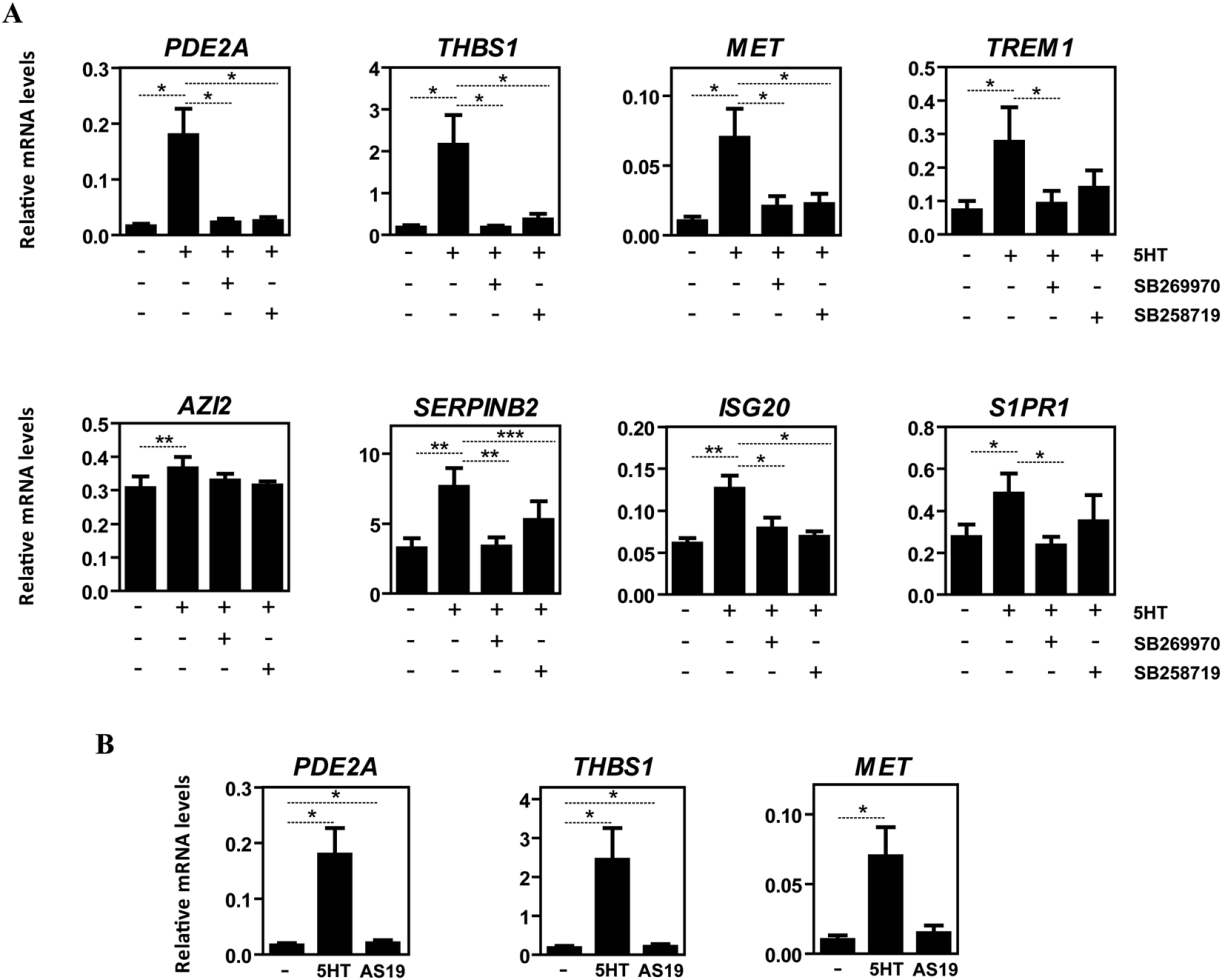
The acquisition of the 5-HT-dependent gene signature of human M-MØ is mediated by the 5-HT7R receptor. (**A**) Relative expression of the indicated genes in non-treated (-) or 5-HT-treated (5HT, 6 h) M-MØ and either in the absence (-) or in the presence of the 5-HT7R antagonists SB269970 or SB258719. Results are expressed as the mRNA level of each gene relative to the *TBP* mRNA level in the same sample (n = 6; *p < 0.05; **p < 0.01; ***p < 0.001). (**B**) Relative expression of the indicated genes in M-MØ either non-treated (-) or treated for 6 h with 5-HT or the 5-HT7R agonist AS19. Results are expressed as the mRNA level of each gene relative to the *TBP* mRNA level in the same sample (n = 6; *p < 0.05).

To determine the receptors responsible for the acquisition of the 5HT-dependent transcriptional and functional profile in 5-HT7R^+^/5-HT2BR^+^ M-MØ^44^, macrophages were exposed to 5-HT in the presence of 5-HT7R antagonists SB269970^50^ or SB258719^51^, or the 5-HT2BR antagonist SB204741^52^. Whereas blockade of 5-HT2BR had no effect (Supplementary Figure 1), 5-HT7R antagonists prevented or significantly inhibited the 5-HT-dependent up-regulation of *PDE2A, THBS1, MET, TREM1*, *SERPINB2, ISG20, AZI2* and *S1PR1* (Fig. 2A). Although to a lower extent than 5-HT, the 5-HT7R agonist AS19 was capable of enhancing the expression of *PDE2A* and *THBS1* (Fig. 2B), further supporting the involvement of 5-HT7R in the 5-HT-dependent gene expression changes in human macrophages. However, since AS19 had no effect on other 5-HT-regulated genes (*MET*) (Fig. 2B), additional receptors might also contribute to the acquisition of the 5-HT-dependent gene signature.

**Figure 3:**
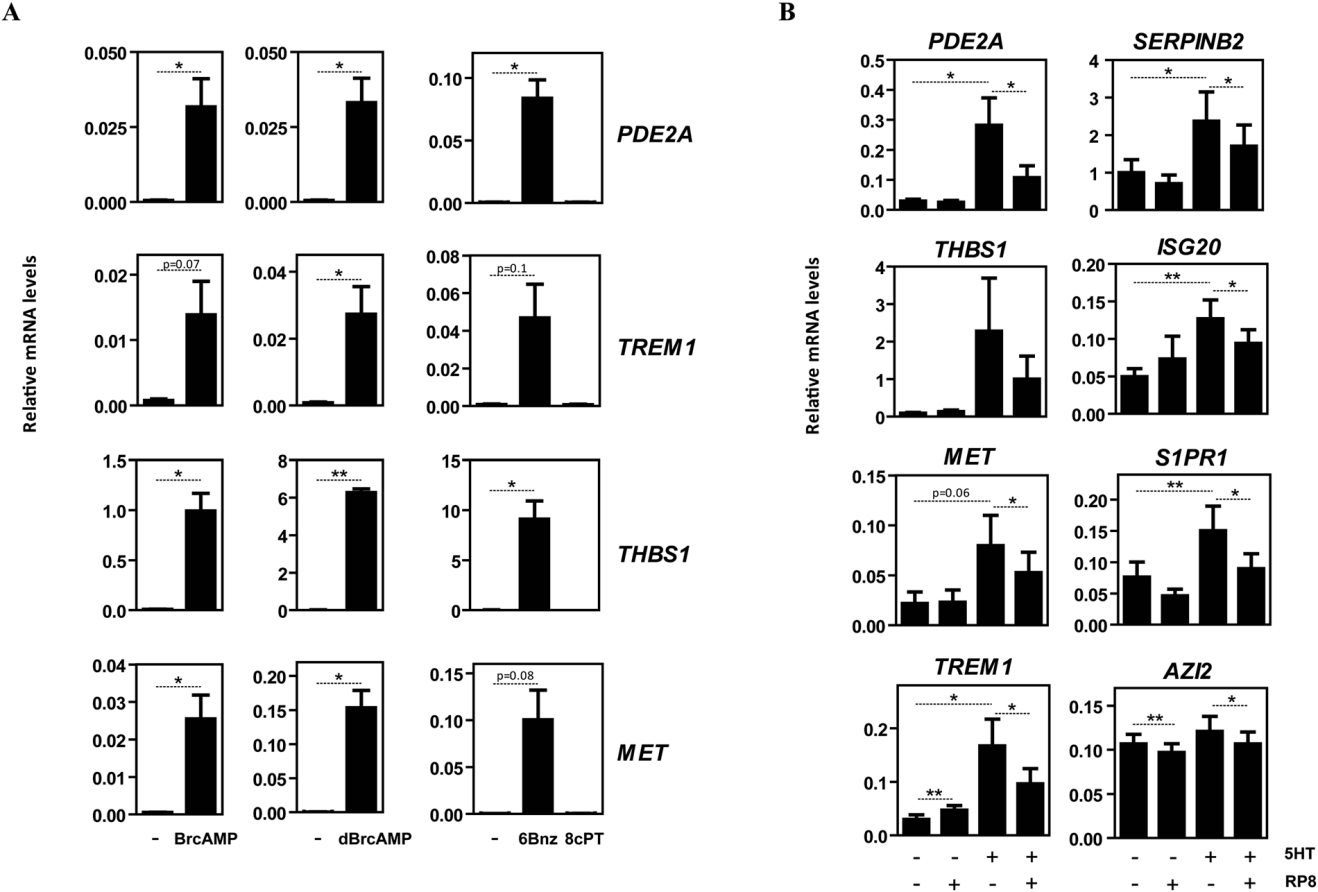
The acquisition of the 5-HT/5-HT7R-dependent gene signature of human M-MØ is mediated by PKA. (**A**) Relative expression of the indicated genes in M-MØ either untreated (-) or treated for 6 h to the PKA activators BrcAMP, dBrcAMP or 6Bnz, or to the Epac activator 8cPT. Results are expressed as the mRNA level of each gene relative to the *GAPDH* mRNA level in the same sample (n = 3; *p < 0.05). (**B**) Relative expression of the indicated genes in non-treated (-) or 5-HT-treated (5HT, 6 h) M-MØ and either in the absence (-) or in the presence of the PKA inhibitor RP8. Results are expressed as the mRNA level of each gene relative to the *TBP* mRNA level in the same sample (n = 6; *p < 0.05; **p < 0.01).

Since 5-HT7R engagement leads to increased intracellular levels of cAMP^53^, whose effectors include PKA and “Exchange factor directly activated by cAMP” (Epac), we next assessed the effect of cAMP analogs (BrcAMP, dBrcAMP) and modifiers of cAMP-initiated signaling (6Bnz, 8cPT, RP8) on the acquisition of the 5-HT-dependent gene signature in M-MØ. The cAMP analogs BrcAMP and dBrcAMP greatly enhanced the expression of genes upregulated by 5-HT (*PDE2A, TREM1, THBS1, MET*) (Fig. 3A). Similar changes in the expression of these genes were seen in M-MØ exposed to the PKA-specific activator 6-Bnz-cAMP (Fig. 3A), while the Epac activator 8-pCPT had no effect (Fig. 3A). Moreover, the positive effect of 5-HT on the expression of 5-HT-upregulated genes was significantly blunted or inhibited in the presence of the PKA inhibitor RP8 (Fig. 3B). Therefore, 5-HT shapes M-MØ gene expression primarily via engagement of 5-HT7R and activation of PKA.

**Figure 4:**
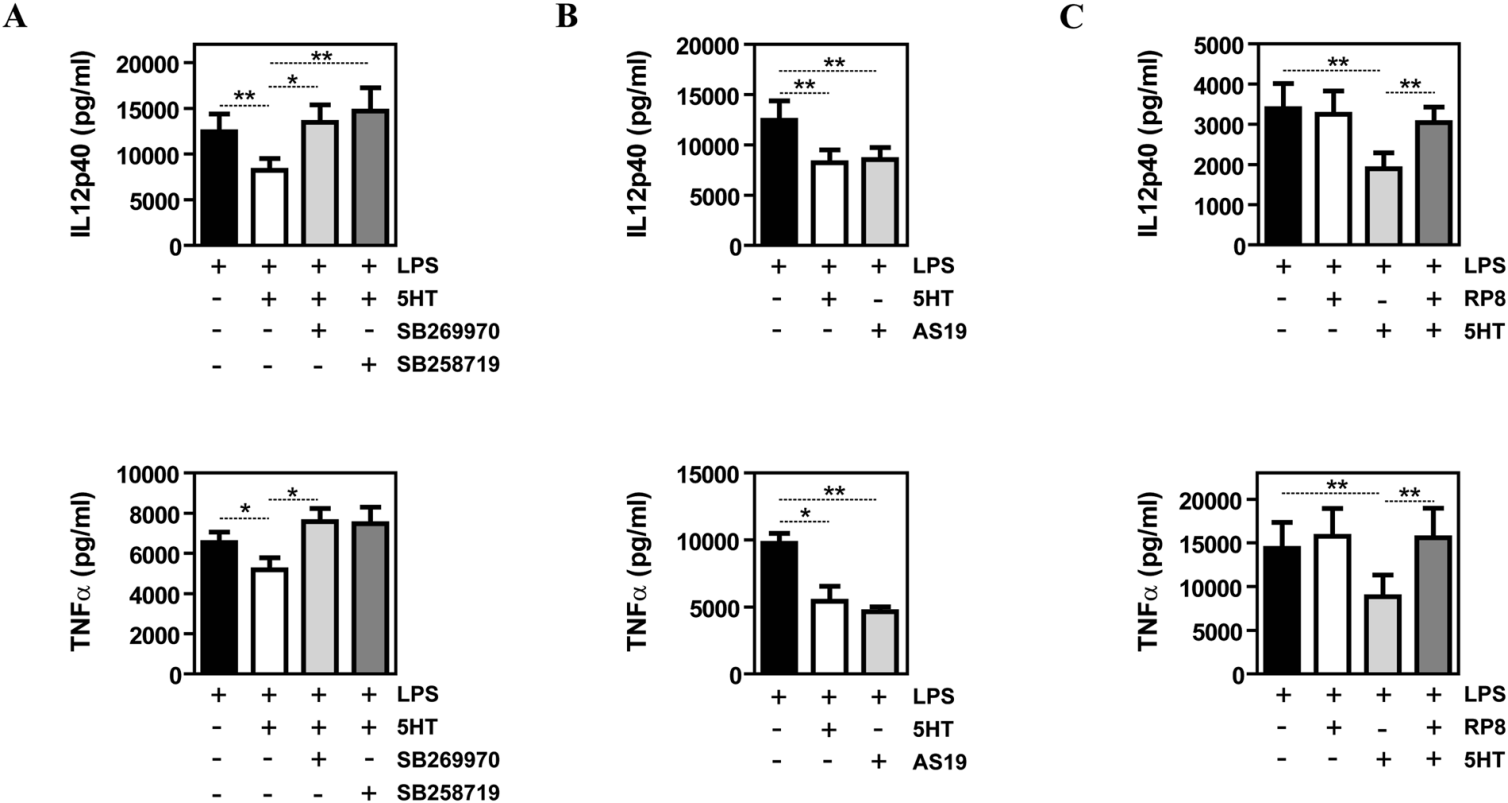
The inhibitory effect of 5-HT on the LPS-induced pro-inflammatory cytokine production of human macrophages is dependent on 5-HT7R and PKA. (**A**) Production of LPS-stimulated IL-12p40 and TNFα by M-MØ non-treated (-) or pretreated with 5-HT (6 h) in the presence or absence of the 5-HT7R antagonists SB269970 or SB258719 (n = 6; *p < 0.05; **p < 0.01; ***p < 0.001). (**B**) Production of LPS-stimulated IL-12p40 and TNFα by M-MØ non-treated (-) or pretreated (6 h) with either 5-HT (5HT) or the 5-HT7R agonist AS19 (n = 6; *p < 0.05; **p < 0.01). (**C**) Production of LPS-stimulated IL-12p40 and TNFα by M-MØ non-treated (-) or pretreated with 5-HT (5HT, 6 h) in the presence or absence of the PKA inhibitor RP8 (n = 6; *p < 0.05; **p < 0.01).

Since the 5-HT7R-PKA axis mediates the expression of the 5-HT-dependent M-MØ transcriptome, we next evaluated whether this signaling axis contributes to the inhibitory effect of 5-HT on the LPS-induced production of inflammatory cytokines by M-MØ, which produce undetectable levels of TNFα and IL-12p40 in the absence of stimulation^44^. Both 5-HT7R antagonists (SB269970 and SB258719) dose-dependently reversed the inhibitory action of 5-HT on the production of TNFα and IL-12p40 induced by LPS (Fig. 4A), while the 5-HT7R agonist AS19 (1 μM) mimicked the effect of 5-HT on the LPS-induced expression of TNFα and IL-12p40 (Fig. 4B). Furthermore, the inhibitory effect of 5-HT on the LPS-induced production of TNFα and IL-12p40 was significantly reduced in the presence of the PKA inhibitor RP8 (Fig. 4C). Thus, 5-HT conditions macrophages for a diminished production of pro-inflammatory cytokines primarily via engagement of 5-HT7R and activation of PKA. As a whole, this set of results demonstrates that the 5-HT7R-PKA axis mediates the acquisition of the 5-HT-dependent gene and cytokine profile in human macrophages.

### 5-HT impairs type I IFN-dependent gene and chemokine expression through the 5-HT7R-PKA axis

**Figure 5:**
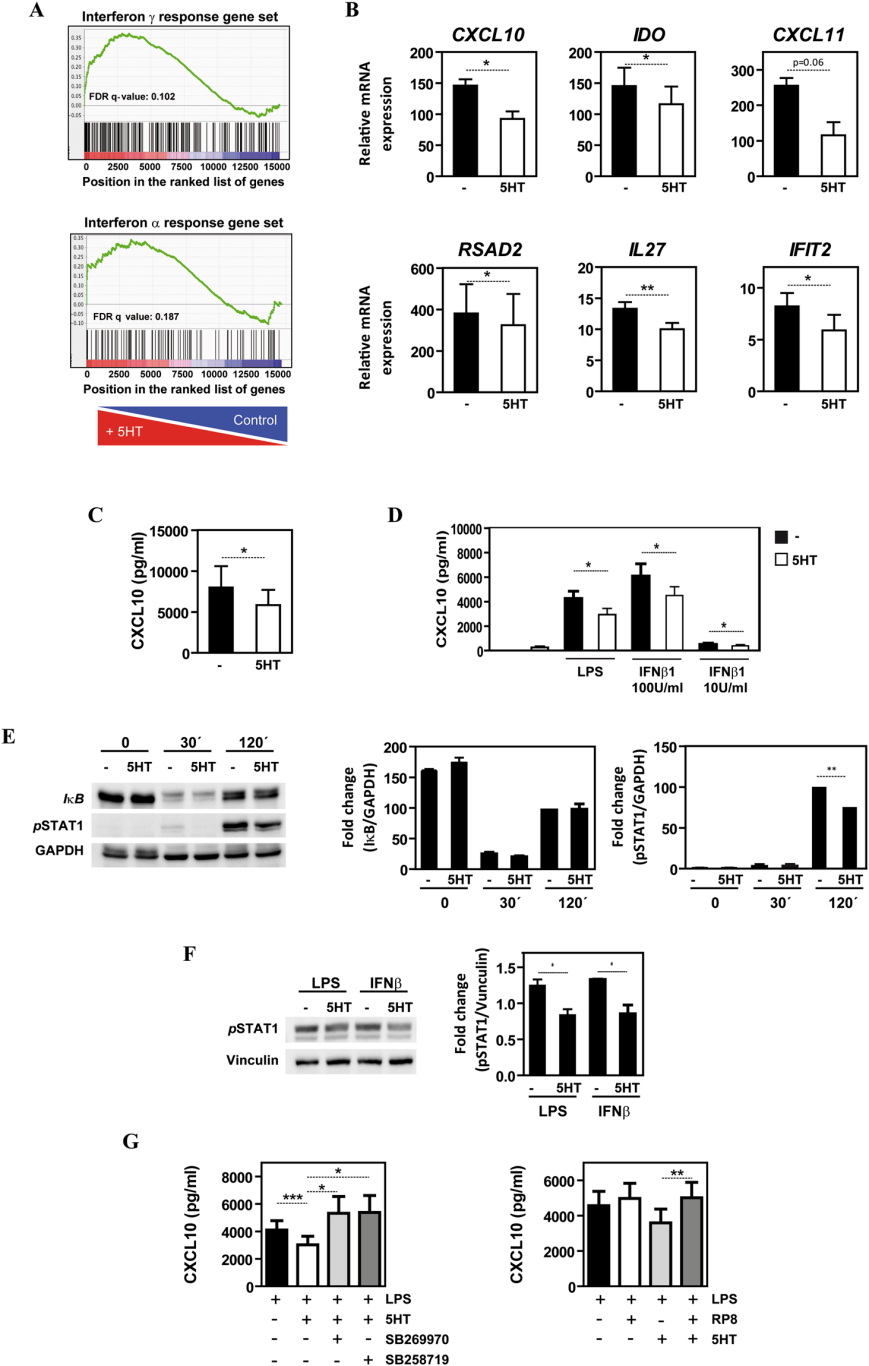
Serotonin modifies the type I IFN-dependent gene profile and impairs the response of human macrophages to type I IFN through 5-HT7R and PKA. (**A**) GSEA on the “t statistic-ranked” list of genes obtained from the 5-HT-treated M-MØ versus M-MØ limma analysis, using the “Hallmark_Interferon_gamma_response” (left panel) and the “Hallmark_Interferon_alpha_response” (right panel) gene sets. (**B**) Expression of the indicated genes in untreated (-) or 5-HT-pretreated (5HT, 6 h) LPS-stimulated (4 h) M-MØ, as determined by qRT-PCR. Results are expressed as the mRNA level of each gene relative to the level of *TBP* mRNA in the same sample (n = 3; *p < 0.05; **p < 0.01). (**C**) Production of LPS-stimulated CXCL10 by untreated (-) M-MØ and 5-HT-treated M-MØ (5HT, 6 h) exposed to LPS for 18 h (n = 12; *p < 0.05). (**D**) Production of IFNβ1-stimulated CXCL10 by M-MØ non-treated (-) or pretreated with 5-HT (5HT, 6 h), using the indicated concentrations of IFNβ1 (n = 13; *p < 0.05). (**E**) Levels of IkBα and phosphorylated STAT1 in untreated (-) M-MØ and 5-HT-treated M-MØ (5HT, 6 h), after stimulation with LPS for the indicated periods of time (left panel). Protein loading was normalized using a monoclonal antibody against GAPDH. Densitometric analysis of three independent experiments is shown in the right panels (n = 3; **p < 0.01). (**F**) Levels of phosphorylated STAT1 in untreated (-) M-MØ and 5-HT-treated M-MØ (5HT, 6 h), after stimulation with LPS or IFNβ1 (for 2 h). Protein loading was normalized using a monoclonal antibody against Vinculin. Densitometric analysis of three independent experiments is shown in the right panels (n = 3; *p < 0.05). (**G**) Production of LPS-stimulated CXCL10 by non-treated (-) M-MØ or M-MØ pretreated with 5-HT (5HT, 6 h) in the presence or absence of the 5-HT7R antagonists SB269970 or SB258719 (n = 6; *p < 0.05; ***p < 0.001) (left panel) or in the presence or absence of the PKA inhibitor RP8 (n = 6; **p < 0.01) (right panel).

To gain further biological insights from the 5-HT-dependent M-MØ transcriptome, we performed GSEA on the 5-HT-induced gene profile using gene sets contained in the Molecular Signature databases available at the GSEA Website. GSEA on the 5-HT-dependent transcriptome using the gene sets revealed a significant enrichment of the “hallmark IFNγ response” (FDR q value = 0.102) and “hallmark IFNα response” (FDR q value = 0.187) gene sets within the genes upregulated by 5-HT (Fig. 5A). In agreement with the GSEA data, the mRNA levels of type I IFN-dependent genes (*CXCL10, CXCL11, IDO1, RSAD2, IL27* and *IFIT2*) (Fig. 5B) and the production of the type I IFN-dependent chemokine CXCL10 (Fig. 5C) were significantly reduced in LPS-treated M-MØ that had been pre-treated for 6 hours with 5-HT. Moreover, M-MØ also produced reduced levels of CXCL10 in response to IFNβ if previously exposed to 5-HT (Fig. 5D). The decrease in LPS- or IFNβ-induced CXCL10 production caused by 5-HT exposure correlated with a reduced activation of STAT1 in response to either LPS or IFNβ (Fig. 5E,F), further supporting the 5-HT´s ability to limit macrophage responses to type I IFN. Importantly, the ability of 5-HT to limit the expression of type I IFN-responsive genes is also mediated by the 5-HT7R-PKA axis, as it was abolished by either 5-HT7R antagonists (SB269970 and SB258719) or the PKA inhibitor RP8 (Fig. 5G).

### 5-HT also promotes pro-fibrotic gene expression in human macrophages

**Figure 6:**
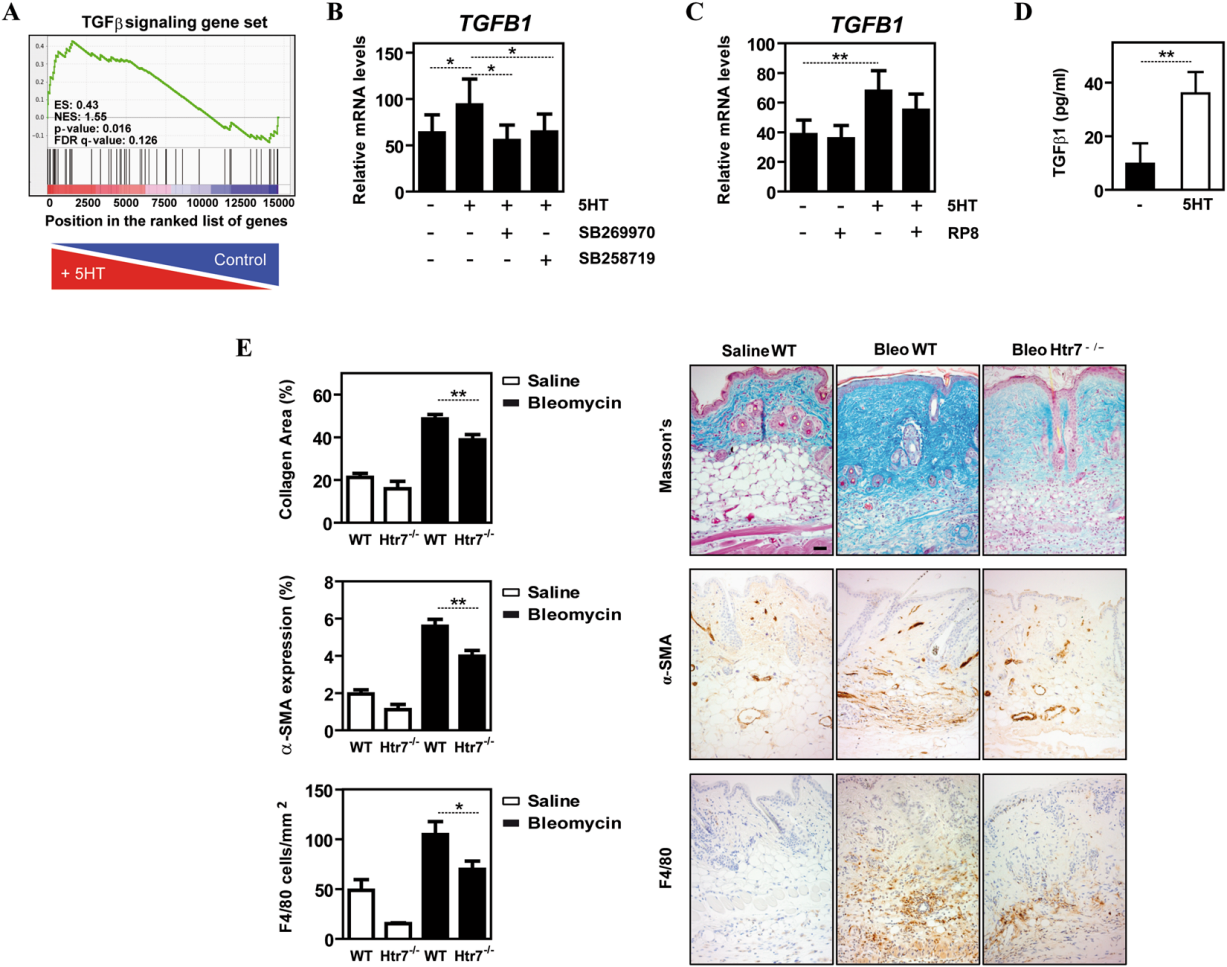
5-HT7R engagement promotes the acquisition of a pro-fibrotic gene signature in human macrophages and contributes to pathology-associated parameters in a mouse model of skin fibrosis. (**A**) GSEA on the “t statistic-ranked” list of genes obtained from the 5-HT-treated M-MØ versus M-MØ limma analysis, using the “TGFβ signaling” gene set. Black vertical lines indicate the position of each of the genes included within the “TGFβ signaling” Hallmark gene set. (**B**) Relative expression of *TGFB1* mRNA in non-treated M-MØ (-) or M-MØ exposed to 5-HT (5HT, 6 h) in the absence or in the presence of the 5-HT7R antagonists SB269970 or SB258719. Results are expressed as the *TGFB1* mRNA level relative to the *TBP* mRNA level in the same sample (n = 6; *p < 0.05). (**C**) Relative expression of *TGFB1* mRNA in non-treated M-MØ (-) or M-MØ exposed to 5-HT (5HT, 6 h) in the absence or in the presence of the PKA inhibitor RP8. Results are expressed as the *TGFB1* mRNA level relative to the *TBP* and *HPRT1* mRNA level in the same sample (n = 6; *p < 0.05; **p < 0.01). (**D**) Production of TGFβ1 by non-treated M-MØ (-) or M-MØ treated with 5-HT (5HT) for 24 h. (n = 3; **p < 0.01). (**E**) Fibrosis was measured as the collagen Masson stained area (upper panel), immunohistochemistry analysis of activated fibroblasts (α-SMA^+^, middle panel) and number of F4/80^+^ cells per area (lower panel), in lesional skin from saline- or bleomycin-treated control and *Htr7* ^−/−^ mice. Shown are the mean and SEM of three independent experiments with 10 mice per group. Statistical significance was evaluated using Mann-Whitney U-test, (*p < 0.05; **p < 0.01). Representative skin sections stained are shown (bar, 50 μM).

Additional GSEA results evidenced that a short-term (6 h) exposure to 5-HT causes a global upregulation of the “Angiogenesis” gene sets, as well as a very significant downregulation of the “Cholesterol homeostasis” and “Fatty Acid Metabolism” genes sets (Supplementary Figure 2). Furthermore, 5-HT treatment led to a significant upregulation of the “TGFβ signaling” gene set (FDR q-val = 0.126) (Fig. 6A). In line with the GSEA data, 5-HT was found to induce a significant increase in *TGFB1* mRNA (Fig. 6B). Like most 5-HT-upregulated genes, the enhanced expression of *TGFB1* mRNA was prevented by 5-HT7R antagonists (Fig. 6B) and did not occur in the presence of the PKA inhibitor RP8 (Fig. 6C). More importantly, 5-HT treatment of M-MØ resulted in a significantly increase production of TGFβ1 (Fig. 6D). Therefore, engagement of 5-HT7R by 5-HT promotes the production of TGFβ1 as well as the acquisition of a pro-fibrotic gene signature in human macrophages.

### Lack of Htr7 results in diminished macrophage infiltration and pathology in a mouse model of skin fibrosis

Macrophages exert critical functions during tissue repair after injury but their deregulated polarization can also result in excessive scarring and chronic fibrosis^54,55^. In fact, macrophages are important cells for the onset of scleroderma^56,57^ and pulmonary fibrosis^58,59^, and their deregulated polarization results in fibrosis in muscle^60^. Given the 5-HT/5-HT7R-upregulated expression of TGFβ1 in human macrophages, and to analyze the contribution of 5-HT7R to skin fibrosis development, we assessed the effect of *Htr7* gene ablation in the mouse model of bleomycin-induced dermal fibrosis that mimics histological features of human scleroderma^61^. To this end we first determined *Htr7* expression of in mouse macrophages *in vitro* and *in vivo*. *Htr7* mRNA was readily detected in liver Kupffer cells and peritoneal F4/80^+^ macrophages, with *Htr7a* being the predominant splicing isoform in both cases (Supplementary Figure 3A). Unlike human monocyte-derived macrophages, *Htr7* mRNA was only detected in murine pro-inflammatory GM-MØ, where also *Htr7a* was the predominant isoform (Supplementary Figure 3B). Also in marked contrast with human macrophages, where *HTR7* mRNA is greatly reduced in response to LPS^44^, macrophage *Htr7* mRNA was greatly upregulated after LPS stimulation (Supplementary Figure 3C).

Once the presence of *Htr7* mRNA had been demonstrated in murine macrophages, the effect of *Htr7* gene deletion in a mouse model of fibrosis was assessed. Whereas no histological differences in collagen stained area were observed between saline-treated *Htr7* ^−/−^ and WT mice, a significant increase in skin collagen content was observed after bleomycin injection in WT mice (Fig. 6E). Conversely, *Htr7* ^−/−^ mice appeared protected from bleomycin-induced fibrosis since significantly reduced collagen area content was observed in bleomycin-treated *Htr7* ^−/−^ skin compared to WT skin (Fig. 6E). In addition, myofibroblast differentiation evaluated as α-SMA expression, was also significantly reduced in *Htr7* ^−/−^ compared to WT mice (Fig. 6E). Likewise, a significantly lower infiltration of F4/80^+^ cells was found in bleomycin-treated *Htr7* ^−/−^ mice (Fig. 6E). In addition, bleomycin treatment significantly enhanced α-SMA expression in WT mice but not in *Htr7* ^−/−^ mice (Fig. 6E). Therefore, and in line with the transcriptional results in human macrophages, 5-HT7R expression contributes to macrophage accumulation and fibrosis in the bleomycin model of skin fibrosis.

## Discussion

Macrophages exhibit a huge phenotypic and functional heterogeneity, and their effector functions (“polarization state”) are determined by the integration of the intracellular signals initiated by the surrounding extracellular cues and stimuli. Elimination of inflammatory insults requires the balanced and sequential dominance of pro-inflammatory and anti-inflammatory/resolving macrophages^62^ whose deregulation leads to chronic inflammatory diseases^63–65^. Given their critical role in the initiation and resolution of inflammation, modulation of the macrophage polarization state has been proposed as a therapeutic approach for numerous chronic inflammatory pathologies^37^. While the physiological processes regulated by 5-HT (cell proliferation, tissue repair, inflammation) are also critically modulated by macrophages^66^, the influence of 5-HT on macrophage plasticity is not yet completely understood^11^. Based on the ability of 5-HT to modulate the macrophage cytokine profile^44^, we undertook the determination of the 5-HT-dependent human macrophage transcriptome. Our results indicate that 5-HT conditions macrophages for impaired production of pro-inflammatory cytokines and type I IFN-inducible cytokines, and also shapes the macrophage gene signature towards the acquisition of an anti-inflammatory and pro-fibrotic gene profile, with all these effects being primarily mediated by the 5-HT7R-PKA axis.

The link between 5-HT and fibroblast proliferation/fibrosis has been known to be primarily mediated by the 5-HT2BR receptor, which induces extra-cellular matrix synthesis in fibroblasts^19^ and whose over-activation leads to excessive proliferation of cardiac valves fibroblasts and severe cardiac pathologies^67–69^. Our results reveal that 5-HT7R is a primary mediator of the pro-fibrotic action of 5-HT on human macrophages because 5-HT7R antagonists and inhibitors of 5-HT7R-initiated signaling block the acquisition of the pro-fibrotic gene signature as well as the 5-HT-upregulated production of TGFβ1 in human macrophages. The pro-fibrotic action of 5-HT7R is further supported by *in vivo* results, as *Htr7* ^−/−^ mice exhibit diminished pathology (lower collagen deposition and F4/80^+^ cell infiltration) in the bleomycin-induced model of skin fibrosis. Macrophages play a critical role in fibrotic processes^54^. In fact, elimination of macrophages expressing Folr2, a myeloid-specific protein exclusively expressed by anti-inflammatory M-MØ^70^, greatly diminishes pathology in the bleomycin-induced experimental skin fibrosis^71^. Therefore, it can be speculated that the absence of 5-HT7R in mouse macrophages contributes to the diminished skin pathology we have observed. Alternatively, other macrophage 5-HT7R-dependent functions might contribute to the reduced pathology seen in *Htr7* ^−/−^ mice. Specifically, impaired migration of myeloid cells to the bleomycin-treated tissue might explain the reduced accumulation of F4/80^+^ cells in the damaged skin of *Htr7* ^−/−^ mice. This explanation would fit with the impaired migration of mouse bone marrow-derived dendritic cells from *Htr7* ^−/−^ mice^72^ and is supported by the significant 5-HT-dependent upregulation of genes involved in cell chemotaxis (Fig. 1D). However, these interpretations should be taken cautiously, because expression of 5-HT7R appears to be differentially regulated in mouse and human macrophages: 5-HT7R in human macrophages is greatly downregulated by pathogenic stimuli like LPS^44^ whereas *Ht7r* expression in mouse myeloid cells is greatly upregulated by LPS^72^. In line with our findings, the 5-HT7R agonist LP-44 has been reported to reduce pro-inflammatory cytokine production *in vivo* in a carbon tetrachloride-induced rat model of liver fibrosis^73^, where the agonist was, however, also capable of inhibiting *Tgfb1* mRNA^73^. The latter discrepancy between these results and ours might be explained by the use of different 5-HT7R agonists (5HT versus a chemical agonist) and animal models of fibrosis (bleomycin-induced mouse skin fibrosis versus carbon tetrachloride-induced rat liver fibrosis), but indicate a significant involvement of 5-HT7R in fibrotic responses.

In peripheral tissues, 5-HT7R is expressed in smooth muscle cells of blood vessels and the gastrointestinal tract, as well as in kidney, liver, pancreas and spleen^74–76^. Within the immune system, the functional consequences of 5-HT7R ligation are far from clear. 5-HT7R on mouse naive T cells contributes to early T-cell activation^12^, whereas 5-HT7R on human monocytes has been reported to inhibit^77^ or enhance^78^ LPS-induced pro-inflammatory cytokine release. A similar controversy appears to exist regarding the *in vivo* role of *Htr7*, whose deletion has been reported to improve^22^ or exacerbate^23^ mouse gut inflammation. Our transcriptional results clarify this issue and demonstrate that 5-HT7R, acting through PKA, conditions macrophages towards the acquisition of a more anti-inflammatory and pro-fibrotic polarization state and for an impaired production of pro-inflammatory functions. As a consequence, our results place 5-HT7R as a potentially relevant molecule for modulation of macrophage effector functions under physiological and pathological settings.

The anti-inflammatory gene profile promoted by 5-HT/5-HT7R is compatible with the known signaling ability of 5-HT7R. Although 5-HT7R has been shown to activate NFκB in monocytes^12,78^, and ERK1/2^79^, Akt^80^, p38MAPK and protein kinase Cε^81^, or the Cdc42-Gα12-SRF axis^82^ in various cell types, 5-HT7R couples positively to adenylate cyclase through activating Gαs, leading to increased cAMP levels and activation of PKA and Epac1/2^74^. These cell-specific differences in 5-HT7R signaling might derive from the presence of distinct splicing isoforms or heterodimerization with other receptors^83,84^. In the case of human macrophages, where the three splicing isoforms can be detected at the mRNA level (data not shown), our results clearly establish PKA, and not Epac1/2, as a major effector of 5-HT7R, as most of the transcriptional actions of 5-HT7R engagement by 5-HT can be abolished by PKA inhibitors and mimicked by PKA activators. Furthermore, the connexion between 5-HT7R and PKA activation fits well with the global anti-inflammatory skewing induced by 5-HT via 5-HT7R because PKA leads to CREB activation, which favours the acquisition of a “M2 polarization state”^62^. In addition, cAMP-initiated signaling limits the effector functions of pro-inflammatory stimuli^85,86^, which is consistent with the reduced production of proinflammatory cytokines seen with 5-HT7R activation.

The ability of 5-HT to promote the acquisition of a pro-fibrotic and anti-inflammatory signature in human macrophages has relevant pathophysiological implications. While normal peripheral blood levels of 5-HT range between 0.7 and 2.5 μM^87–91^, 5-HT concentrations at the neuronal synapse have been estimated to reach the millimolar range^92^, and the available platelet serotonin is close to 20 μM^93^. Since platelets release serotonin during inflammation as a means to activate endothelial cells and promote leukocyte adhesion and recruitment^93^, our findings on the macrophage polarizing effects of 10 μM 5-HT are physiologically relevant, especially at the initial stages of inflammatory responses. Besides, and regarding pathology, the serum levels of 5-HT detected in metastatic carcinoid tumors exceed 30 μM^91^, thus pointing to 5-HT as a factor that contributes to polarization of macrophages in serotonin-producing neuroendocrine tumors. Therefore, 5HT7R-regulated genes should be considered as potential targets to modify macrophage polarization under pathological settings.

## Materials and Methods

### Ethical statement

Ethical approvals for all blood sources and processes used in this study have been approved by the Centro de Investigaciones Biológicas Ethics Committees. Subjects gave written informed consent in accordance to the Declaration of Helsinki. All experiments were carried out in accordance with the approved guidelines and regulations.

All experiments on mice were conducted according to the Spanish and European regulations on care and protection of laboratory animals and were approved by the Consejo Superior de Investigaciones Científicas ethics committee.

### Generation of human monocyte-derived macrophages and cell isolation and culture

Human peripheral blood mononuclear cells (PBMC) were isolated from buffy coats from normal donors over a Lymphoprep (Nycomed Pharma, Oslo, Norway) gradient according to standard procedures. Monocytes were purified from PBMC by magnetic cell sorting using CD14 microbeads (Miltenyi Biotech, Bergisch Gladbach, Germany). Monocytes (>95% CD14^+^ cells) were cultured at 0.5 × 10^6^ cells/ml for 7 days in RMI supplemented with 10% fetal calf serum (FCS) (completed medium), at 37 °C in a humidified atmosphere with 5% CO_2_, and containing M-CSF (10 ng/ml) (ImmunoTools GmbH, Friesoythe, Germany) to generate monocyte-derived macrophages (M-MØ). Cytokine was added every two days. Before treatment with 5HT, M-MØ macrophages were maintained in serum-free medium for 48 hours, without a significant change in the level of expression of M-MØ-specific markers. Macrophage activation was accomplished with either ultrapure LPS (*E. coli* 055:B5, 10 ng/ml, Invivogen, San Diego, CA), synthetic triacylated lipoprotein (Pam3CSK4, 10 μg/ml, Invivogen) or IFNγ (10–100 IU/ml, Miltenyi Biotech). Bone marrow-derived mouse macrophages were also generated using GM-CSF (mouse GM-MØ) or M-CSF (mouse M-MØ) as described previously^94,95^. RNA from mouse liver cells was obtained as previously described^44^. Isolation of mouse peritoneal mouse macrophages was done by magnetic cell sorting using F4/80-biotin and Streptavidin microbeads (Miltenyi Biotech). For activation, macrophages were treated with Escherichia coli 055:B5 LPS (100 ng/ml for mouse macrophages; 10 ng/ml for human macrophages) for 24 h. 5-HT was obtained from Sigma Aldrich and used at 10μM. 5-HT7R agonists AS19 was obtained from Tocris Bioscience and used at 1μM. 5-HT7R antagonists (SB269970 and SB258719 were purchased from Sigma Aldrich and Tocris Bioscience, respectively, and added at 10μM 1 hour before 5-HT treatment. cAMP analogues (BrcAMP and dBrcAMP) were used at 200μM and 50μM, respectively. The PKA activator 6BNZ-cAMP (6Bnz) and the EPAC-specific activator 8-pCPT-2′-O-Me-cAMP (8cPT) were obtained from Sigma Aldrich and used at 200μM and 100μM, respectively. The PKA-specific inhibitor RP-8-CPT-cAMPs (RP8) was obtained from Biolog and used at 100μM.

### ELISA

Culture supernatants from untreated or LPS-treated (24 h) human macrophages were assayed for the presence of cytokines using commercially available ELISA for human TNFα, IL-12p40 (BD Pharmingen), CXCL10, IL-10 (Biolegend), activin A and TGFβ (R&D Systems). ELISA was performed following the protocols supplied by the manufacturers.

### Quantitative real-time RT-PCR

Oligonucleotides for selected genes were designed according to the Universal Probe Roche library system (Roche Diagnostics) for quantitative real time PCR (qRT-PCR). Total RNA was extracted using the RNeasy kit (Qiagen), retrotranscribed, and amplified in triplicates. Results were expressed relative to the expression level of *TBP* RNA. When indicated, results were expressed relative to the mean of the expression level of endogenous reference genes HPRT1, TBP and RPLP0. In all cases the results were expressed using the ΔΔCT method for quantitation.

### Microarray analysis

Global gene expression analysis was performed on RNA obtained from untreated or 5-HT-treated (10 μM, 6 h) M-MØ from three independent healthy donors. RNA was isolated using the RNeasy Mini kit (Qiagen, Germantown, MD) and labeled RNA used as a hybridization probe on Whole Human Genome Microarrays (Agilent Technologies, Palo Alto, CA). Only probes with signal values > 60% quantile in at least one condition were considered for the differential expression and statistical analysis. Statistical analysis for differential gene expression was carried out using empirical Bayes moderated t test implemented in the limma package (http://www.bioconductor.org). The p values were further adjusted for multiple hypotheses testing using the Benjamini-Hochberg method to control the false discovery rate^96^. All the above procedures were coded in R (http://www.r-project.org). Microarray data were deposited in the Gene Expression Omnibus (http://www.ncbi.nlm.nih.gov/geo/) under accession GSE94608. Differentially expressed genes were analysed for annotated gene sets enrichment using the online tool ENRICHR (http://amp.pharm.mssm.edu/Enrichr/)^97,98^. Enrichment terms were considered significant when they had a Benjamini-Hochberg-adjusted *p* value < 0.05. For gene set enrichment analysis (GSEA)^99^, the gene sets contained in the Molecular Signature databases available at the GSEA website, and the previously defined “Pro-inflammatory gene set” and “Anti-inflammatory gene set”^48^, which contain the top and bottom 150 probes from the GM-MØ versus M-MØ limma analysis of the microarray data in GSE68061 (ranked on the basis of the value of the t statistic), were used.

### Western blot

Cell lysates were obtained in RIPA buffer (10 mM Tris-HCl pH 8, 150 mM NaCl, 1% NP-40, 2 mM Pefabloc, 2 mg/ml aprotinin/antipain/leupeptin/pepstatin, 10 mM NaF, and 1 mM Na_3_VO_4_). 20 µg of cell lysate was subjected to SDS-PAGE and transferred onto an Immobilon polyvinylidene difluoride membrane (Millipore). Protein detection was carried out using antibodies against phosphorylated STAT1 (BD Biosciences, CA, USA) and a monoclonal antibody against GAPDH (sc-32233, Santa Cruz, CA, USA).

### Bleomycin-induced skin fibrosis mouse model

Skin fibrosis was induced in 6–8 week-old, pathogen-free WT or *Htr7* ^−/−^^100^ female C57BL/6NJ (The Jackson Laboratory) by subcutaneous injections of 100 μg of bleomycin (1 mg/ml, Mylan Pharmaceuticals, Barcelona, Spain) or 0.9% saline control into the shaved back skin every day for 4 weeks as previously described^61,101^. Skin was harvested and frozen for mRNA/protein studies or paraffin embedded for histological studies. Fibrosis was determined by Masson’s trichrome staining, and the presence of α-SMA^+^ or F4/80^+^ determined using anti-α Smooth Muscle Actin antibody (α-SMA; 1A4 clone, Sigma-Aldrich, Spain) and anti-F4/80 antibody (BM8 clone; eBioscience, San Diego, CA, USA).

### Statistical analysis

For comparison of means, and unless otherwise indicated, statistical significance of the generated data was evaluated using the Student t test. In all cases, *p* < 0.05 was considered as statistically significant.

### Data Availability

All data generated or analysed during this study are included in this published article. Moreover, microarray data were deposited in the Gene Expression Omnibus (http://www.ncbi.nlm.nih.gov/geo/) under accession GSE94608.

## Electronic supplementary material

Supplementary Information
