# Supplementary material for: Serotonin drives the acquisition of a profibrotic and anti-inflammatory gene profile through the 5-HT7R-PKA signaling axis
Source: Sci Rep. 2017 Nov 7;7:14761. doi: 10.1038/s41598-017-15348-y (PMC5676747; doi:10.1038/s41598-017-15348-y)
Supplement: Supplementary file 1 — Supplementary Information [file 41598_2017_15348_MOESM1_ESM.pdf]

Serotonin drives the acquisition of a profibrotic and anti-inflammatory gene profile through the 5-HT7R-PKA signaling axis

Ángeles Domínguez-Soto*, Alicia Usategui , Mateo de las Casas-Engel , Miriam Simón-Fuentes, Concha Nieto , Víctor D. Cuevas , Miguel A. Vega , José Luis Pablos , and Ángel L. Corbí.

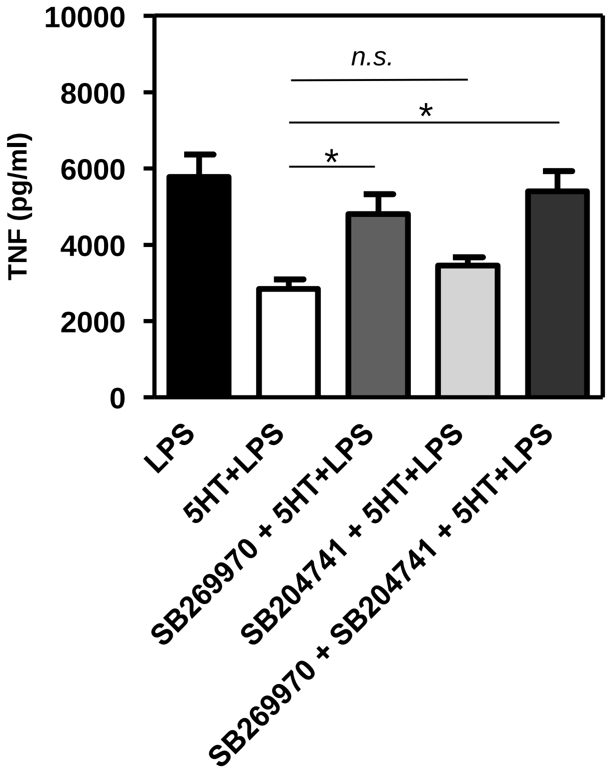

Supplementary Figure 1

Upregulated	FDR q-val
Hallmark Angiogenesis	0.129
Hallmark Hypoxia	0.2
Hallmark Inflammatory response	0.216
Hallmark DNA repair	0.228
Hallmark Glycolysis	0.238

Downregulated	FDR q-val
Hallmark Cholesterol homeostasis	0.0
Hallmark Fatty Acid metabolism	0.012
Hallmark Androgen response	0.015
Hallmark Oxydative phosphorylation	0.018
Hallmark mTORC1 signaling	0.103
Hallmark Myc targets	0.103
Hallmark Estrogen Response late	0.112

Supplementary Figure 2

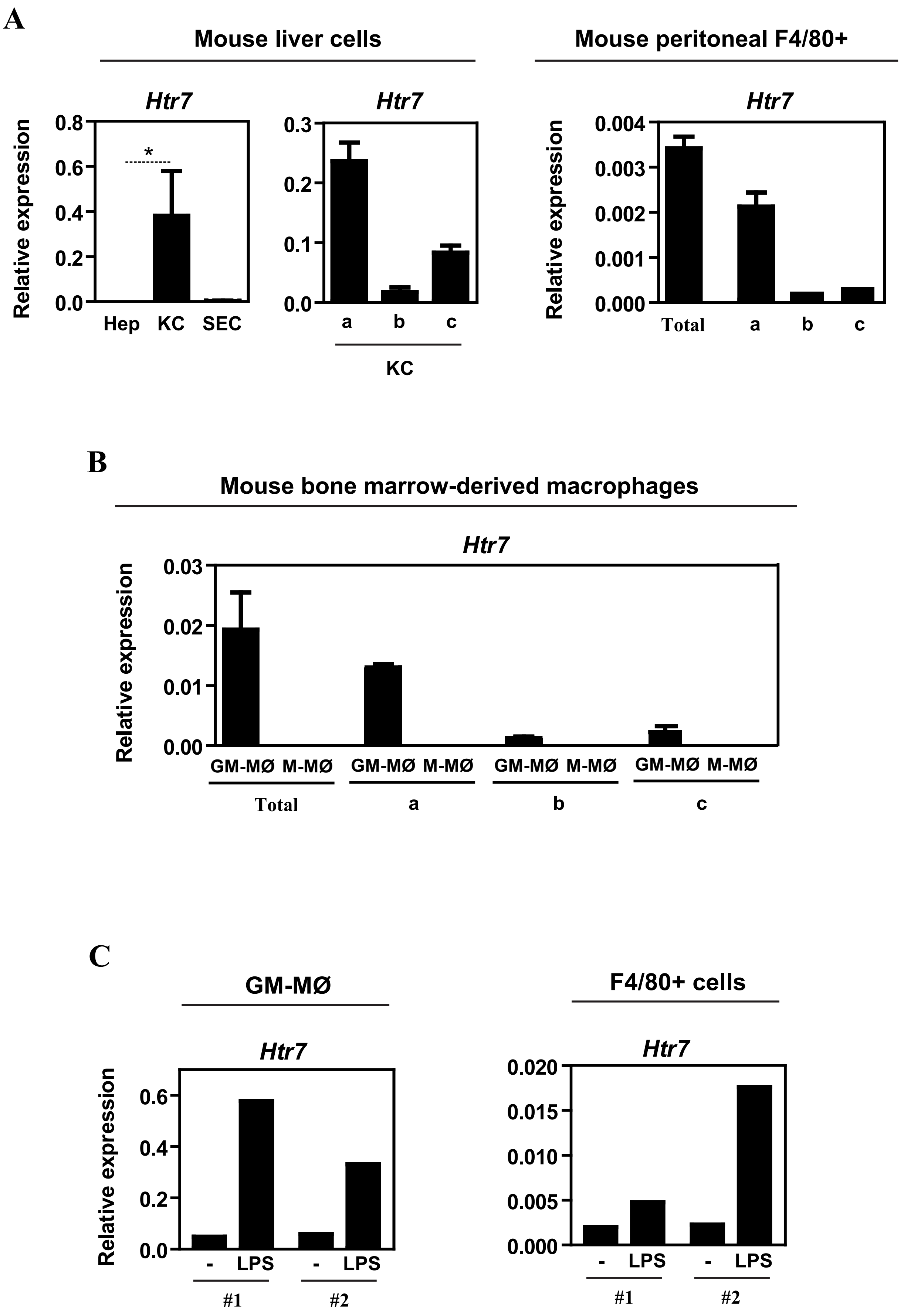

Supplementary Figure 3

Supplementary Table I							
probeid	M-MØ	M-MØ+5HT	log2.fold M-MØ+5HT/M-MØ	t	pval	adj.pval	genesymbol
A_23_P401106	154.79	2069.16	3.68	14.6249	0	0.192	PDE2A
A_23_P19333	80.05	344.74	2.08	16.5371	0	0.192	TREM1
A_24_P349547	218.04	750.66	1.97	9.8847	2.0000E-04	0.2138	
A_33_P3322288	101.13	372.80	1.95	9.6901	2.0000E-04	0.2138	AZI2
A_23_P68031	99.87	385.92	1.92	5.8015	0.0024	0.2918	STAT4
A_23_P359245	36.35	139.40	1.78	4.6261	0.0062	0.3294	MET
A_33_P3262694	146.55	434.36	1.76	6.2628	0.0017	0.2733	
A_33_P3236267	228.15	690.87	1.74	6.4271	0.0015	0.2733	KCNQ1OT1
A_23_P76969	785.27	2508.70	1.60	7.4097	8.0000E-04	0.2392	SIPA1L1
A_23_P132515	102.95	300.13	1.55	12.9583	1.0000E-04	0.2092	SIDT1
A_23_P170453	4108.14	10205.73	1.49	7.968	6.0000E-04	0.2193	CST5
A_33_P3257150	930.53	2405.95	1.45	5.6793	0.0026	0.3013	CDC42
A_24_P179013	29.76	82.16	1.44	4.446	0.0072	0.3315	
A_33_P3372727	54.20	141.80	1.44	6.8087	0.0012	0.2668	SEMA5A
A_23_P416711	51.15	137.60	1.44	14.5716	0	0.192	ST6GALNAC3
A_23_P41344	22.46	64.52	1.43	4.6314	0.0061	0.3294	EREG
A_33_P3422294	101.01	288.34	1.42	7.6364	7.0000E-04	0.2392	
A_24_P245379	51.84	148.73	1.41	8.8935	3.0000E-04	0.2138	SERPINB2
A_23_P201979	541.13	1528.58	1.40	4.4274	0.0074	0.3315	CREM
A_23_P208900	275.45	686.62	1.33	14.3717	0	0.192	SEMA6B
A_23_P428129	407.37	1022.43	1.32	8.6251	4.0000E-04	0.2138	CDKN1C
A_33_P3317431	1153.70	2714.24	1.31	7.5237	8.0000E-04	0.2392	
A_23_P72117	446.16	1126.78	1.30	8.8485	4.0000E-04	0.2138	SMPDL3A
A_23_P102731	127.71	316.19	1.28	8.6233	4.0000E-04	0.2138	SMOX
A_23_P85453	45.04	102.33	1.26	10.2964	2.0000E-04	0.2138	CD244
A_24_P32085	269.48	651.45	1.26	10.2497	2.0000E-04	0.2138	MOB3B
A_33_P3294133	870.49	1877.56	1.25	5.9258	0.0022	0.2918	EIF4B
A_23_P32404	410.56	963.29	1.24	13.1219	1.0000E-04	0.2092	ISG20
A_23_P145529	31.66	75.46	1.20	6.385	0.0016	0.2733	PKIB
A_33_P3308115	990.35	1966.88	1.18	5.2919	0.0035	0.311	
A_23_P257649	331.87	745.56	1.18	12.2964	1.0000E-04	0.2138	RBP1
A_23_P154605	3414.30	7447.51	1.16	10.6682	2.0000E-04	0.2138	SULF2
A_24_P49260	120.84	254.30	1.14	11.5085	1.0000E-04	0.2138	SPTLC3
A_23_P419714	19.58	42.63	1.14	9.2799	3.0000E-04	0.2138	BTBD11
A_24_P677642	30.48	61.09	1.14	5.2716	0.0036	0.311	FLJ31813
A_33_P3407230	27.08	57.92	1.13	5.0078	0.0044	0.3173	
A_23_P404481	116.49	243.45	1.11	8.5894	4.0000E-04	0.2138	S1PR1
A_33_P3258977	35.31	72.61	1.11	8.2306	5.0000E-04	0.2193	CLEC4D
A_33_P3333436	78.19	169.81	1.10	9.9911	2.0000E-04	0.2138	SGSM3
A_23_P373724	55.60	115.94	1.10	11.7738	1.0000E-04	0.2138	PPFIBP1
A_33_P3323722	2078.85	4415.23	1.10	7.9589	6.0000E-04	0.2193	ARL4C
A_23_P79518	166.63	375.23	1.10	7.111	0.001	0.2435	IL1B
A_24_P23245	1433.40	2910.16	1.10	5.095	0.0041	0.3145	NDUFA6
A_33_P3412716	154.06	380.19	1.08	5.0541	0.0043	0.3168	
A_23_P25503	2952.57	6154.31	1.06	8.4164	5.0000E-04	0.2138	FNDC3A
A_33_P3382560	7622.06	15383.62	1.06	8.424	4.0000E-04	0.2138	RPL23A
A_24_P759477	36.54	77.03	1.05	8.6302	4.0000E-04	0.2138	ITGB8
A_33_P3229390	496.15	987.05	1.05	5.6149	0.0027	0.3038	
A_33_P3275055	18.28	36.20	1.03	6.2679	0.0017	0.2733	ST6GALNAC3

A_24_P291826	173.38	346.79	1.02	5.8185	0.0023	0.2918	SYTL3
A_32_P4985	21.76	43.44	1.01	5.5752	0.0028	0.3038	CAMTA1
A_33_P3273885	409.11	870.16	1.01	7.9645	6.0000E-04	0.2193	
A_32_P84605	14673.78	28072.13	1.00	4.8911	0.0049	0.3196	RPS23
A_33_P3319905	23.59	46.75	0.99	10.9177	1.0000E-04	0.2138	TREM1
A_33_P3366053	276.73	534.19	0.99	5.9429	0.0021	0.2918	ADPRH
A_33_P3395314	647.87	1272.55	0.99	5.3382	0.0034	0.3095	
A_23_P144959	43.39	85.50	0.98	10.5655	2.0000E-04	0.2138	VCAN
A_23_P166297	227.26	450.70	0.98	8.6544	4.0000E-04	0.2138	ABCG1
A_23_P74278	162.12	299.62	0.98	4.4996	0.0069	0.3315	PDE4B
A_32_P117464	190.00	365.73	0.97	7.6492	7.0000E-04	0.2392	MB21D2
A_23_P251075	111.82	212.26	0.97	4.835	0.0051	0.3231	MAMLD1
A_23_P414654	56.39	113.19	0.96	8.9112	3.0000E-04	0.2138	RAB37
A_23_P23438	741.59	1400.56	0.96	7.4906	8.0000E-04	0.2392	SEMA4A
A_24_P62505	31.16	59.65	0.96	4.9374	0.0047	0.3196	COLGALT2
A_23_P59637	957.90	1874.73	0.96	7.9163	6.0000E-04	0.2193	DOCK4
A_24_P296508	702.06	1395.79	0.95	8.7928	4.0000E-04	0.2138	SLC43A2
A_24_P381604	1672.70	2917.42	0.95	5.128	0.004	0.3145	ITM2B
A_23_P321354	389.53	744.31	0.95	10.037	2.0000E-04	0.2138	TMEM71
A_24_P270460	30.36	63.95	0.94	6.9762	0.0011	0.2531	IFI27
A_23_P60565	62.44	113.57	0.94	5.3352	0.0034	0.3095	ZNF354A
A_24_P132383	491.79	927.06	0.94	9.3149	3.0000E-04	0.2138	GIMAP8
A_33_P3352578	74.65	140.30	0.93	10.043	2.0000E-04	0.2138	CLEC4D
A_23_P39550	38.95	68.20	0.92	4.7259	0.0056	0.3294	TMEM163
A_23_P571	246.24	421.15	0.91	4.9355	0.0047	0.3196	SLC2A1
A_33_P3413840	121.69	228.70	0.91	6.8214	0.0012	0.2668	GK
A_33_P3273919	33.02	62.25	0.90	5.8288	0.0023	0.2918	RNF212
A_23_P91850	27.53	52.53	0.90	5.0045	0.0044	0.3173	IL20RB
A_23_P31858	98.63	174.64	0.90	4.9561	0.0046	0.3196	ST18
A_33_P3240843	170.64	314.95	0.88	8.4384	4.0000E-04	0.2138	TMEM71
A_23_P214139	349.66	647.84	0.88	7.2258	9.0000E-04	0.2392	REV3L
A_23_P69109	1470.47	2684.51	0.87	9.957	2.0000E-04	0.2138	PLSCR1
A_32_P161762	69.56	124.60	0.87	8.7471	4.0000E-04	0.2138	RUNX2
A_23_P208334	48.63	87.49	0.87	7.2443	9.0000E-04	0.2392	PDE4A
A_23_P68601	54.52	97.95	0.87	6.3335	0.0016	0.2733	CST7
A_33_P3375934	679.07	1219.59	0.86	5.0331	0.0043	0.3173	NAMPT
A_23_P81760	25.62	45.18	0.86	6.4934	0.0014	0.2733	QKI
A_33_P3389653	22.84	39.46	0.85	4.6774	0.0059	0.3294	PDE4D
A_23_P167920	117.43	211.91	0.85	7.7999	6.0000E-04	0.2296	DLL1
A_23_P66432	239.80	437.52	0.85	5.49	0.003	0.3082	TTYH2
A_23_P425502	53.16	94.05	0.84	6.4028	0.0015	0.2733	DONSON
A_23_P24077	2990.99	5175.59	0.84	6.2178	0.0018	0.2772	C10orf54
A_24_P315184	9659.77	16932.61	0.83	7.7041	7.0000E-04	0.2387	NBEAL1
A_23_P115011	59.66	102.35	0.83	7.3499	8.0000E-04	0.2392	ADAMTSL4
A_33_P3259557	296.93	544.75	0.83	7.1249	0.001	0.2435	TMEM198B
A_33_P3279456	1040.31	1927.41	0.82	5.8947	0.0022	0.2918	
A_23_P55356	279.95	489.77	0.82	7.2414	9.0000E-04	0.2392	VMO1
A_24_P93703	371.19	656.60	0.82	8.9764	3.0000E-04	0.2138	TMEM198B
A_24_P141332	475.35	791.34	0.82	4.2302	0.0088	0.3318	CAMK2G
A_23_P55649	43.70	76.22	0.81	7.4754	8.0000E-04	0.2392	FPR2
A_33_P3287348	460.32	754.77	0.81	5.3999	0.0032	0.3095	CHN2
A_24_P417606	8592.71	14024.86	0.81	4.3835	0.0077	0.3318	
A_23_P110445	129.59	225.31	0.81	7.5107	8.0000E-04	0.2392	APBB3

A_23_P400378	512.19	844.84	0.81	6.8421	0.0011	0.2668	GPBAR1
A_33_P3559138	690.61	1148.55	0.81	5.3174	0.0034	0.3095	
A_24_P413126	50.01	87.03	0.80	7.4007	8.0000E-04	0.2392	PMEPA1
A_23_P38795	1232.70	2077.44	0.80	6.7376	0.0012	0.2692	FPR1
A_33_P3226050	548.01	953.06	0.80	8.4776	4.0000E-04	0.2138	GATSL3
A_23_P91390	1169.22	2064.74	0.80	5.3811	0.0033	0.3095	THBD
A_23_P201747	114.55	205.24	0.79	6.4307	0.0015	0.2733	PADI2
A_33_P3232557	119.84	206.66	0.78	6.5667	0.0014	0.2733	DLGAP3
A_33_P3415191	182.14	313.76	0.78	6.6105	0.0013	0.2733	ATP8B1
A_24_P324674	492.95	846.92	0.78	8.9556	3.0000E-04	0.2138	LY9
A_23_P86653	11484.56	19458.03	0.77	8.3019	5.0000E-04	0.2193	SRGN
A_23_P15108	532.44	913.10	0.77	8.1222	5.0000E-04	0.2193	YPEL3
A_23_P89570	551.51	948.42	0.76	8.0314	6.0000E-04	0.2193	ZMYND15
A_33_P3353259	19313.92	31984.65	0.76	5.2107	0.0038	0.3134	
A_33_P3238685	85.84	142.42	0.76	4.8244	0.0052	0.3237	TET2
A_24_P397817	26.80	49.85	0.76	4.5795	0.0064	0.3315	LEP
A_23_P210253	455.64	781.74	0.76	5.7366	0.0025	0.298	DGKD
A_33_P3421695	672.37	1171.67	0.76	4.1928	0.0091	0.3318	CTNNB1
A_24_P33156	80.86	135.90	0.75	4.889	0.0049	0.3196	AFMID
A_23_P10559	264.88	447.64	0.75	8.4521	4.0000E-04	0.2138	AATK
A_23_P152791	163.03	270.51	0.75	6.851	0.0011	0.2668	SLC16A6
A_23_P37375	217.10	359.90	0.74	8.0574	5.0000E-04	0.2193	RPS6KA5
A_23_P380998	165.10	269.18	0.74	6.4024	0.0015	0.2733	R3HDM1
A_33_P3227716	820.46	1369.66	0.73	7.4591	8.0000E-04	0.2392	GATSL3
A_24_P189739	38.88	64.36	0.73	4.5853	0.0064	0.3315	DUSP16
A_23_P154849	87.31	145.08	0.73	7.9088	6.0000E-04	0.2193	OLIG1
A_23_P258108	11895.64	19363.83	0.73	6.3718	0.0016	0.2733	
A_33_P3294524	90.88	148.39	0.72	5.3412	0.0034	0.3095	ANKRD12
A_23_P42116	264.29	453.39	0.72	4.936	0.0047	0.3196	PPT2
A_24_P58620	89.92	144.32	0.72	4.1773	0.0093	0.3318	GRAMD1B
A_24_P370172	236.53	391.70	0.72	7.0777	0.001	0.2457	LILRA5
A_23_P53588	24.99	41.18	0.72	5.2053	0.0038	0.3134	WNT5B
A_33_P3390708	37.54	64.61	0.72	4.439	0.0073	0.3315	TMEM2
A_33_P3242623	865.18	1305.65	0.72	4.3188	0.0081	0.3318	SLC7A11
A_32_P222961	30.14	49.40	0.72	7.198	9.0000E-04	0.2392	SPIN4
A_33_P3290955	5568.64	9146.74	0.71	5.3543	0.0033	0.3095	KIAA1875
A_23_P353316	31.74	50.36	0.71	5.2575	0.0036	0.311	SMURF1
A_33_P3328619	6678.08	11020.05	0.71	4.7319	0.0056	0.3294	
A_23_P215744	91.56	144.62	0.71	6.3883	0.0016	0.2733	CTTNBP2
A_23_P25566	4726.99	7713.17	0.71	6.3212	0.0016	0.2733	GPR183
A_33_P3258988	17.45	29.52	0.71	4.9046	0.0048	0.3196	GDI2
A_23_P166508	549.11	889.84	0.70	5.2813	0.0035	0.311	
A_33_P3236177	703.17	1139.93	0.70	7.9894	6.0000E-04	0.2193	ANG
A_24_P261383	202.16	310.89	0.70	4.2333	0.0088	0.3318	TAF1D
A_23_P85716	2690.03	4201.95	0.69	6.8414	0.0011	0.2668	FCGR2A
A_23_P371011	48.04	75.06	0.69	5.4183	0.0032	0.3095	ZNF227
A_24_P54131	247.63	380.98	0.69	4.5339	0.0067	0.3315	DCLRE1B
A_23_P99625	27.67	44.38	0.69	4.9355	0.0047	0.3196	FITM1
A_33_P3351775	79.06	130.10	0.69	7.4673	8.0000E-04	0.2392	
A_24_P342807	23.75	39.53	0.69	5.9989	0.0021	0.2918	SLC30A6
A_32_P87697	29475.87	46802.86	0.68	7.3421	8.0000E-04	0.2392	HLA-DRA
A_24_P313418	68.68	118.09	0.68	5.7423	0.0025	0.298	CCL22
A_24_P237586	275.01	447.66	0.68	5.0103	0.0044	0.3173	ANKRD37

A_33_P3263666	2204.73	3570.41	0.68	4.4619	0.0071	0.3315	ANKRD9
A_23_P110196	173.85	287.18	0.68	5.9138	0.0022	0.2918	HERC5
A_33_P3295333	26.76	42.54	0.68	5.155	0.0039	0.3134	CLEC1A
A_33_P3236392	249.82	382.87	0.67	5.8762	0.0022	0.2918	PVRL4
A_23_P2745	17.93	29.77	0.67	5.7744	0.0024	0.2953	GJB6
A_23_P402319	337.02	507.54	0.67	5.1038	0.0041	0.3145	GAPT
A_24_P477051	23.51	37.44	0.67	6.1669	0.0018	0.2816	
A_24_P288993	162.96	270.55	0.66	4.2925	0.0083	0.3318	
A_33_P3386132	397.26	623.42	0.66	6.3502	0.0016	0.2733	C2orf49
A_23_P10077	443.79	705.56	0.66	6.2905	0.0017	0.2733	PNPLA2
A_24_P22079	225.45	352.04	0.66	5.9146	0.0022	0.2918	FOXO1
A_23_P57760	101.41	159.07	0.66	7.4768	8.0000E-04	0.2392	ACPL2
A_24_P212819	3107.33	4788.86	0.65	6.2808	0.0017	0.2733	
A_24_P390060	219.39	346.68	0.65	5.6028	0.0028	0.3038	IQCD
A_24_P195724	1585.50	2347.11	0.65	4.3066	0.0082	0.3318	
A_23_P24044	47.34	73.82	0.65	7.3834	8.0000E-04	0.2392	CNNM2
A_23_P37359	168.99	253.57	0.65	5.0175	0.0044	0.3173	CIDEB
A_23_P49499	279.35	414.74	0.65	4.651	0.006	0.3294	ST6GALNAC2
A_23_P82128	17218.41	26359.21	0.64	5.9828	0.0021	0.2918	RPS12
A_33_P3234472	60.21	97.99	0.64	5.1275	0.004	0.3145	LOC284751
A_33_P3417589	25.18	40.08	0.64	4.3282	0.0081	0.3318	
A_33_P3352098	11435.65	17831.64	0.64	6.7192	0.0012	0.2692	MS4A7
A_23_P60166	395.34	627.24	0.64	6.3945	0.0016	0.2733	DEPTOR
A_33_P3284883	48.11	74.30	0.64	6.3034	0.0017	0.2733	MOB3B
A_24_P110273	676.36	1044.92	0.63	6.3217	0.0016	0.2733	
A_33_P3327799	3260.03	5077.60	0.63	5.9058	0.0022	0.2918	
A_23_P26024	4892.64	7101.14	0.63	4.5079	0.0068	0.3315	C15orf48
A_33_P3272090	154.84	239.22	0.63	4.7594	0.0055	0.3288	PDE4DIP
A_33_P3295650	61.03	95.87	0.62	5.5974	0.0028	0.3038	APBA1
A_32_P135818	7584.21	11366.12	0.62	5.2381	0.0037	0.3121	RPS3A
A_23_P217428	62.29	97.58	0.62	5.0527	0.0043	0.3168	ARHGAP6
A_24_P332341	227.80	367.39	0.62	4.3021	0.0082	0.3318	POLR2J4
A_33_P3382031	70.26	108.03	0.62	6.2503	0.0017	0.2736	TMEM236
A_23_P9883	1124.10	1674.39	0.62	5.326	0.0034	0.3095	NLRP3
A_23_P41114	878.89	1323.40	0.62	6.4491	0.0015	0.2733	CSTA
A_23_P55020	656.97	1013.28	0.61	6.659	0.0013	0.2725	CD300LF
A_23_P422851	605.75	902.87	0.61	5.8119	0.0024	0.2918	CABLES1
A_23_P140848	324.38	493.00	0.61	4.9716	0.0046	0.3196	MPHOSPH6
A_24_P63136	107.12	161.75	0.61	6.2917	0.0017	0.2733	P2RY13
A_24_P363548	31.41	47.43	0.61	6.2105	0.0018	0.2772	HIP1
A_23_P70398	47.75	70.80	0.61	4.3648	0.0078	0.3318	VEGFA
A_33_P3216427	523.77	813.73	0.61	4.8868	0.0049	0.3196	DGKQ
A_33_P3367994	385.69	568.55	0.60	5.8335	0.0023	0.2918	TAF8
A_24_P48408	98.56	142.17	0.60	4.5112	0.0068	0.3315	RNMT
A_32_P217750	49.31	78.60	0.60	4.2311	0.0088	0.3318	IL3RA
A_33_P3315320	70.38	106.96	0.60	6.7149	0.0012	0.2692	CNTD1
A_23_P64343	2067.77	3068.07	0.60	5.7079	0.0025	0.3005	TIMM10
A_23_P343398	58.18	85.73	0.60	5.3014	0.0035	0.3105	CCR7
A_23_P62967	1743.65	1144.21	-0.60	-6.7737	0.0012	0.2689	DISC1
A_33_P3317168	418.46	276.28	-0.60	-4.5822	0.0064	0.3315	EPS8L3
A_23_P372096	49.48	33.26	-0.60	-4.1053	0.0099	0.3318	C20orf112
A_23_P18119	83.37	52.02	-0.61	-4.2356	0.0088	0.3318	IMPG2
A_23_P125204	492.06	317.00	-0.61	-6.4729	0.0015	0.2733	OR10G8

A_33_P3371109	51.32	33.28	-0.62	-5.701	0.0026	0.3005	LOC100130597
A_33_P3404097	2144.26	1411.17	-0.63	-5.0479	0.0043	0.3169	PGM5P2
A_33_P3380850	47.45	30.57	-0.63	-7.2047	9.0000E-04	0.2392	PAX2
A_24_P376787	499.87	337.10	-0.64	-4.73	0.0056	0.3294	ZNF496
A_23_P146284	2607.18	1715.24	-0.64	-4.2267	0.0088	0.3318	SQLE
A_33_P3315824	105.26	67.94	-0.64	-4.5628	0.0065	0.3315	
A_33_P3381827	432.23	287.12	-0.65	-4.5535	0.0066	0.3315	OSBPL2
A_32_P134290	1591.48	1022.82	-0.65	-6.7639	0.0012	0.2689	ZCCHC2
A_23_P150903	261.74	167.82	-0.65	-5.3154	0.0035	0.3095	FAR2
A_33_P3297978	21265.37	13729.91	-0.65	-5.547	0.0029	0.3038	MYO1E
A_24_P587443	38.51	23.78	-0.66	-4.2511	0.0086	0.3318	
A_23_P340848	1536.35	979.80	-0.67	-7.0574	0.001	0.246	PTGIR
A_23_P48784	34.89	21.22	-0.67	-4.8737	0.005	0.3196	CYP46A1
A_33_P3310296	406.56	256.02	-0.67	-5.7208	0.0025	0.2994	
A_33_P3242039	594.73	354.94	-0.67	-4.1768	0.0093	0.3318	
A_23_P500400	118.89	76.68	-0.67	-4.5846	0.0064	0.3315	ABCA6
A_23_P32006	45.87	28.63	-0.68	-7.1463	9.0000E-04	0.2435	OR13D1
A_33_P3324786	283.86	169.49	-0.69	-4.8484	0.0051	0.3212	
A_23_P250619	2274.92	1342.70	-0.72	-5.3412	0.0034	0.3095	ZDHHC14
A_33_P3281468	55.49	33.39	-0.73	-4.8617	0.005	0.3197	STARD9
A_33_P3397348	136.33	80.84	-0.73	-4.7319	0.0056	0.3294	LOC149134
A_33_P3512350	675.48	399.65	-0.76	-6.9789	0.0011	0.2531	LOC339807
A_33_P3214314	1141.14	713.42	-0.76	-4.3225	0.0081	0.3318	CCSER2
A_23_P81441	1493.93	874.24	-0.76	-8.7105	4.0000E-04	0.2138	C5orf20
A_23_P328545	440.40	259.98	-0.76	-8.0807	5.0000E-04	0.2193	GABRP
A_33_P3274134	777.16	461.18	-0.77	-4.3718	0.0077	0.3318	TMEM151B
A_33_P3232955	920.03	555.00	-0.77	-6.662	0.0013	0.2725	F2RL3
A_33_P3263867	5610.72	3207.27	-0.79	-4.7778	0.0054	0.3285	P2RX7
A_23_P97394	1334.01	788.36	-0.80	-5.3565	0.0033	0.3095	BCAR3
A_33_P3380383	4374.93	2579.19	-0.81	-8.8739	4.0000E-04	0.2138	TIFAB
A_23_P58009	32.28	18.15	-0.82	-8.8114	4.0000E-04	0.2138	C3orf52
A_23_P34345	116.89	68.07	-0.83	-4.8014	0.0053	0.3267	VCAM1
A_23_P91095	523.44	291.00	-0.84	-9.6298	2.0000E-04	0.2138	CD28
A_23_P103034	214.84	114.56	-0.91	-7.2742	9.0000E-04	0.2392	CRYBA4
A_23_P67896	269.49	137.51	-0.92	-6.3894	0.0016	0.2733	SCN3A
A_33_P3393655	103.21	52.60	-0.93	-5.5633	0.0028	0.3038	LGI2
A_23_P390116	389.38	221.87	-0.98	-4.4755	0.007	0.3315	SPATA13
A_32_P155666	716.76	350.13	-1.07	-9.703	2.0000E-04	0.2138	ECEL1
A_23_P404162	100.99	46.26	-1.08	-8.2829	5.0000E-04	0.2193	HDAC9
A_33_P3304998	197.28	94.61	-1.09	-4.7524	0.0055	0.3291	LOC101930176
A_24_P41706	77.88	21.11	-1.81	-7.2246	9.0000E-04	0.2392	OFCC1

SUPPLEMENTARY FIGURES

Supplementary Figure 1. Lack of effect of 5-HT2BR blockade on the inhibitory effect of 5-HT on the LPS-induced TNFα production. Production of LPS-stimulated TNFα by M-MØ non-treated or pretreated with 5-HT (6 h) in the presence or absence of the 5-HT7R antagonist SB269970 (10 μM) or the 5-HT2BR antagonist SB204741 (10 μM) (n=3; *, $p < 0.05$; **, $p < 0.01$; ns, non-significant).

Supplementary Figure 2. GSEA Hallmark gene sets significantly regulated upon M-MØ exposure to 5-HT. Results of GSEA on the "t statistic-ranked" list of genes obtained from the 5-HT-treated M-MØ versus M-MØ limma analysis, and using the "Hallmark gene sets" contained in the Molecular Signature databases available at the GSEA website. The gene sets significantly upregulated or down-regulated in the transcriptome of 5HT-treated M-MØ are shown. The False Discovery Rate (FDR q-val) for each GSEA is indicated.

Supplementary Figure 3. Htr7 mRNA expression in *ex vivo*-isolated and *in vitro*-generated mouse macrophages. A. Relative expression of the Htr7 mRNA and Htr7 splicing isoforms (a,b,c) in mouse liver cells (Hep, Hepatocytes; KC, Kupffer Cells; SEC, Sinusoidal Endothelial Cells) and mouse peritoneal F4/80$^+$ (n=3, *, $p < 0.05$). B. Relative expression of the Htr7 mRNA and Htr7 splicing isoforms (a,b,c) in mouse bone marrow-derived GM-MØ and M-MØ (n=3). C. Relative Htr7 mRNA expression in untreated (-) or LPS-stimulated mouse bone marrow-derived GM-MØ (upper panel) and peritoneal F4/80$^+$ cells (lower panel). Two independent experiments were performed and both are shown. In all cases (A-C), results are expressed as the Htr7 mRNA level relative to the Tbp mRNA level in the same sample.

Supplementary Table I. Gene expression analysis on untreated or 5-HT-treated (10 μM, 6h) M-MØ. List of 170 upregulated annotated genes ($p<0.01$; log2 ratio 5-HT/untreated ≥ 0.6) and 41 downregulated genes ($p<0.01$; log2 ratio 5-HT/untreated ≤ -0.6).
